# lncHUB2: aggregated and inferred knowledge about human and mouse lncRNAs

**DOI:** 10.1093/database/baad009

**Published:** 2023-03-04

**Authors:** Giacomo B Marino, Megan L Wojciechowicz, Daniel J B Clarke, Maxim V Kuleshov, Zhuorui Xie, Minji Jeon, Alexander Lachmann, Avi Ma’ayan

**Affiliations:** Department of Pharmacological Sciences, Department of Artificial Intelligence and Human Health, Mount Sinai Center for Bioinformatics, Icahn School of Medicine at Mount Sinai, One Gustave L. Levy Place, Box 1603, New York, NY 10029, USA; Department of Pharmacological Sciences, Department of Artificial Intelligence and Human Health, Mount Sinai Center for Bioinformatics, Icahn School of Medicine at Mount Sinai, One Gustave L. Levy Place, Box 1603, New York, NY 10029, USA; Department of Pharmacological Sciences, Department of Artificial Intelligence and Human Health, Mount Sinai Center for Bioinformatics, Icahn School of Medicine at Mount Sinai, One Gustave L. Levy Place, Box 1603, New York, NY 10029, USA; Department of Pharmacological Sciences, Department of Artificial Intelligence and Human Health, Mount Sinai Center for Bioinformatics, Icahn School of Medicine at Mount Sinai, One Gustave L. Levy Place, Box 1603, New York, NY 10029, USA; Department of Pharmacological Sciences, Department of Artificial Intelligence and Human Health, Mount Sinai Center for Bioinformatics, Icahn School of Medicine at Mount Sinai, One Gustave L. Levy Place, Box 1603, New York, NY 10029, USA; Department of Pharmacological Sciences, Department of Artificial Intelligence and Human Health, Mount Sinai Center for Bioinformatics, Icahn School of Medicine at Mount Sinai, One Gustave L. Levy Place, Box 1603, New York, NY 10029, USA; Department of Pharmacological Sciences, Department of Artificial Intelligence and Human Health, Mount Sinai Center for Bioinformatics, Icahn School of Medicine at Mount Sinai, One Gustave L. Levy Place, Box 1603, New York, NY 10029, USA; Department of Pharmacological Sciences, Department of Artificial Intelligence and Human Health, Mount Sinai Center for Bioinformatics, Icahn School of Medicine at Mount Sinai, One Gustave L. Levy Place, Box 1603, New York, NY 10029, USA

## Abstract

Long non-coding ribonucleic acids (lncRNAs) account for the largest group of non-coding RNAs. However, knowledge about their function and regulation is limited. lncHUB2 is a web server database that provides known and inferred knowledge about the function of 18 705 human and 11 274 mouse lncRNAs. lncHUB2 produces reports that contain the secondary structure fold of the lncRNA, related publications, the most correlated coding genes, the most correlated lncRNAs, a network that visualizes the most correlated genes, predicted mouse phenotypes, predicted membership in biological processes and pathways, predicted upstream transcription factor regulators, and predicted disease associations. In addition, the reports include subcellular localization information; expression across tissues, cell types, and cell lines, and predicted small molecules and CRISPR knockout (CRISPR-KO) genes prioritized based on their likelihood to up- or downregulate the expression of the lncRNA. Overall, lncHUB2 is a database with rich information about human and mouse lncRNAs and as such it can facilitate hypothesis generation for many future studies. The lncHUB2 database is available at https://maayanlab.cloud/lncHUB2.

**Database URL**: https://maayanlab.cloud/lncHUB2

## Introduction

Most of the transcribed genome encodes non-coding ribonucleic acids (ncRNAs) compared with protein-coding genes (1). ncRNAs were once assumed to have no function and were referred to as ‘junk RNA’ due to their lack of protein product. However, it was soon realized that ncRNAs play critical roles in functional and regulatory eukaryotic biology (2). Interestingly, it has been shown that the number of ncRNAs exponentially increases with organismal complexity (3). Most evidence so far established that ncRNAs play an important role in regulating gene expression (4), while many ncRNAs were implicated as key factors in a broad range of diseases (5). Long non-coding RNAs (lncRNAs), defined as ncRNAs having >200 nucleotides in length, account for the largest portion of ncRNAs. However, knowledge about their function is still limited. lncRNAs have been shown to directly interact with proteins, deoxyribonucleic acid (DNA), as well as other RNA molecules, highlighting their potential involvement in the formation of macromolecular complexes and participation in many biological processes (6). It is also established that lncRNAs play an important role in cell differentiation and development (7). Additionally, lncRNAs have been suggested as disease biomarkers due to their stability (8). lncRNAs have been associated with diseases such as cardiovascular disease, neurological disorders, and various cancers (9, 10). Despite this rapid progress, only few lncRNAs have well-established roles where their function, localization, and membership in biological processes, pathways, and diseases have been elucidated. As the number of lncRNA–disease associations has increased in recent years, the interest in their potential role to serve as drug targets has also increased (10). Currently, there are few RNA-based therapeutics including small interfering RNAs (siRNAs) and antisense oligonucleotides (ASOs) that bind to RNAs in a sequence-specific manner (11, 12). Due to the ability of lncRNAs to form secondary structures and their ability to interact with their targets in a structure-specific manner, small molecules can also target lncRNAs due to their ability to disrupt lncRNA–target interactions (13). Small molecules are also less costly to produce and easier to deliver than siRNAs or ASOs (14). However, siRNAs or ASOs have the advantage of being more specific while requiring much less effort, time, and cost to identify and develop.

To fill the knowledge gap that currently exists in our understanding of the roles of human and mouse lncRNAs, there has been an increase in the development of digital resources that consolidate information about lncRNAs. For example, the Rfam database compiles sequence and structure information from the literature to create multiple sequence alignments, secondary structure, and covariance models for thousands of ncRNA families, which facilitate ncRNA DNA/RNA sequence annotation (15). lncBOOK (16) is a web-based resource that serves curated knowledge about human lncRNAs including conservation, variation, methylation, expression, interactions, and disease associations. Similarly, LNCipedia (17) is a web server resource that provides data from manual curation of publications about lncRNAs. Both databases provide functional knowledge about ∼3000 human lncRNAs directly curated from the literature.

Several other databases curate lncRNA associations with diseases, targets and biological functions manually from the literature, e.g., LncRNADisease 2.0 (18), Lnc2Cancer 3.0 (19), LincSNP 3.0 (20), LncTarD (21), and LncACTdb 3.0 (22). On the other hand, LncRNA2Function (23) and Co-LncRNA (24) are web server applications that provide inferred knowledge about lncRNAs based on RNA-seq co-expression data. Extending this idea, Lnc-GFP (25) and LncRNAs2Pathways (26) integrate co-expression data with protein–protein interaction data and employ graph theory algorithms to predict gene function for human lncRNAs. Furthermore, LnCompare (27) integrates additional features such as gene structure and evolutionary conservation to improve predictions. Fewer resources provide information about lncRNA/small-molecule associations. For example, LncTarD provides associations between lncRNAs and drug targets (21). LNCmap identified groups of lncRNAs perturbed by 1262 small molecules using the Connectivity Map (CAMP) database (28), and enrichment analysis to link diseases to these drugs (29). D-lnc reanalyzed 7037 microarray gene expression datasets from the Gene Expression Omnibus (30) and the CMAP database to associate differentially expressed lncRNAs in response to drug perturbations and predicted lncRNA–drug interactions using lncRNA sequence similarity and drug structure similarity (31). Nevertheless, both LNCmap and D-lnc are limited by their relatively low lncRNA coverage. Recently, gene–gene co-expression correlations were used to expand lncRNA coverage (32). In their study Wang et al. prioritized drugs to modulate cancer-associated lncRNAs by computing the overlap between differentially expressed genes (DEGs) for each drug in CMAP with lncRNA-associated genes found via co-expression correlations computed for different cancer types in The Cancer Genome Atlas (33). However, neither of these studies leveraged the availability of the LINCS L1000 data (34), which contain >3 million expression profiles for >30 000 small molecules (35). The LINCS L1000 dataset is a major expansion to the original CMAP. To summarize the collection of lncRNA knowledge bases and resources and to compare these with the information provided by lncHUB2, we organized key common features across these resources in a comprehensive table (Table 1).

**Table 1.** Comparison of features from resources providing information or analysis relating to lncRNAs

Resource	PMID	URL	A	B	C	D	E	F	G	H	I	J	K	L	M
lncHUB2		https://maayanlab.cloud/lncHUB2/	✓	✕	✓	✓	✓	✕	✕	✓	✓	✓	✓	✓	✓
lncBOOK	30715521	https://ngdc.cncb.ac.cn/lncbook/	✕	✓	✕	✕	✕	✓	✓	✕	✕	✕	✕	✕	✓
LNCipedia	30371849	https://lncipedia.org/	✕	✓	✕	✕	✕	✓	✕	✓	✓	✓	✓	✕	✓
LncRNA2Function	25707511	http://mlg.hit.edu.cn/lncrna2function	✕	✕	✕	✓	✓	✕	✕	✕	✕	✕	✕	✕	✕
Co-LncRNA	26363020	http://bio-bigdata.hrbmu.edu.cn/Co-LncRNA/	✕	✕	✕	✓	✓	✕	✕	✕	✕	✕	✕	✕	✕
FANTOM6	32718982	https://fantom.gsc.riken.jp/6/	✕	✓	✓	✓	✕	✕	✕	✕	✕	✕	✕	✓	✓
LnCompare	31147707	http://www.rnanut.net/lncompare/	✓	✓	✕	✕	✓	✕	✕	✕	✕	✕	✕	✓	✓
lncATLAS	28386015	https://lncatlas.crg.eu/	✓	✕	✕	✓	✕	✕	✕	✕	✕	✕	✕	✓	✕
LncTarD	31713618	http://bio-bigdata.hrbmu.edu.cn/LncTarD1.0/	✕	✕	✓	✕	✓	✕	✕	✕	✕	✕	✕	✕	✓
LncRNADisease	30285109	http://www.rnanut.net/lncrnadisease/	✕	✕	✕	✕	✓	✕	✕	✓	✕	✕	✕	✕	✓
Lnc2Cancer	33219685	http://www.bio-bigdata.com/lnc2cancer/	✕	✓	✓	✕	✓	✓	✓	✕	✕	✕	✕	✕	✓
LncSNP	33219 661	http://bioinfo.hrbmu.edu.cn/LincSNP	✕	✓	✕	✕	✓	✕	✕	✕	✕	✕	✕	✕	✕
LncACTdb	34850125	http://www.bio-bigdata.net/LncACTdb/	✕	✕	✓	✕	✓	✕	✕	✕	✕	✕	✕	✓	✓
LNCmap	29325141	http://bio-bigdata.hrbmu.edu.cn/LncMAP/	✕	✕	✓	✕	✓	✕	✕	✕	✕	✕	✕	✕	✓
D-lnc	31390943	http://www.jianglab.cn/D-lnc/	✕	✕	✓	✓	✕	✕	✕	✕	✕	✕	✕	✕	✓
Lnc-GFP	23132350	N/A	✕	✕	✕	✕	✓	✕	✕	✕	✕	✕	✕	✕	✕
LncRNAs2Pathways	28425476	https://cran.r-project.org/web/packages/LncPath/	✕	✕	✕	✕	✓	✕	✕	✕	✕	✕	✕	✕	✓

If a resource had a broken URL, its features were taken from the relevant literature. Column values are as follows: A: expression across tissues, B: variants, C: drugs, D: co-expressed genes, E: function predictions, F: conservation, G: methylations, H: literature, I: structure, J: sequence, K: API, L: subcellular localization, and M: URL to site works.

Here, we introduce lncHUB2, a database and an Appyter, that produces reports with knowledge about the function and regulation of 18 705 human and 11 274 mouse lncRNAs inferred from RNA-seq gene–gene co-expression correlations. lncHUB2 gene page reports provide knowledge about the predicted structure of the lncRNA, related publications, most correlated coding and non-coding genes, predicted biological processes, regulation by transcription factors, disease associations, average expression across tissues and cell lines, cellular localization, and predicted small molecules and CRISPR knock-outs (KOs) of single genes to up-/down-regulate the expression of the lncRNA based on the LINCS L1000 data. Overall, lncHUB2 is a comprehensive resource that bridges the knowledge gap between lncRNAs, diseases, biological functions, and small molecules at the genome-wide scale.

## Results

### The lncHUB2 Appyter and database: serving lncRNA gene page reports

lncHUB2 is implemented as an Appyter and as a full-stack web-based application with a user interface and a backend database. Appyters (36) are light-weight bioinformatics applications directly created from Jupyter Notebooks (37). A collection of Appyters that perform various types of bioinformatics data analysis pipelines are hosted on the Appyters Catalog. The lncHUB2 Appyter produces reports about human lncRNAs by fetching knowledge from multiple sources and making predictions about lncRNA functions via gene–gene co-expression correlations. To make predictions, the lncHUB2 Appyter utilizes a gene–gene co-expression matrix generated from RNA-seq data downloaded from ARCHS4 (38) to generate predictions for 18 705 unique human and 11 274 unique mouse lncRNAs. For each lncRNA, the lncHUB2 Appyter generates a report in the form of tables and interactive and static visualizations, which are available for download as CSV, HTML and static PNG, SVG and PDF files. The lncHUB2 Appyter results were precomputed for the annotated lncRNAs and are stored in the lncHUB2 database. In addition to the Appyter, a user interface provides access to the database via a landing page for each lncRNA. Both the lncHUB2 web-based application and Appyter take as input a human Ensembl ID (39) or a human GENCODE lncRNA name (40) and generate a report for each human lncRNA. The lncHUB2 user interface has an additional functionality that enables users to submit genomic co-ordinates to search for lncRNAs within a specific genomic region.

**Figure 1.**
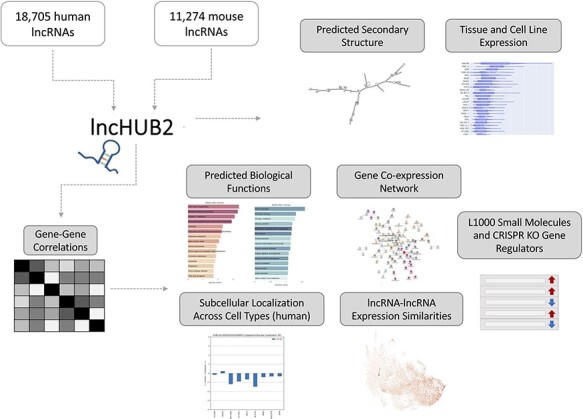
lncHUB2 Appyter and web application workflow. The lncHUB2 Appyter or web-based application takes as input 18 705 unique human and 11 274 unique mouse lncRNAs and generates a report. This report contains useful information such as the predicted secondary structure and expression levels in various tissues and cell lines. Additionally, using gene–gene correlations generated from publicly available RNA-seq data from ARCHS4, lncHUB2 provides predicted biological functions, as well as predicted small molecules and CRISPR-KO gene regulators, and gene-gene co-expression networks to explore closely related genes and lncRNAs associations based on expression similarity.

Once a qualified lncRNA identifier is submitted to the lncHUB2 database, the user is redirected to the corresponding results landing page report (Figure 1). At the top of the report, the predicted secondary structure of the lncRNA is visualized with RNAfold (41), an RNA folding tool that is based on a thermodynamics algorithm. The predicted secondary structure can be downloaded as a PNG file by clicking a download button. The top of the landing page also contains canonical and alternative transcript sequences for the input lncRNA, which were extracted from Ensembl (42). These sequences can be downloaded as CSV files. Next, the landing page displays the frequency of publications for the input lncRNA from 1992 to 2021. The PubMed IDs (PMIDs) and dates can be downloaded as a CSV file. Next, the landing page displays tables containing the top positively and negatively correlated coding genes, and the top positively and negatively correlated lncRNAs with the input lncRNA. Correlations are computed with the Pearson correlation coefficient (PCC) using the most recent version of ARCHS4 V2 (38) processed with the kallisto aligner (43) against GENCODE V41, which corresponds to Ensembl 107. To visualize the top positive gene–gene correlations, an interactive network, made of the top 100 most correlated genes with the submitted lncRNA, is produced. Each node in the network represents a gene, and nodes are colored based on their chromosomal origin with the exception for the lncRNA in focus, which is colored in red. The edges that connect the nodes in the network represent correlation levels. Clicking on a node highlights its edges. Hovering over a node displays both the gene name and its chromosomal location. Next, links to Enrichr (44), a comprehensive gene-set enrichment analysis tool, are available for the top 25, 50, 100, 200, 300 and 500 positively and negatively most correlated genes with the input lncRNA. For each gene-set library in Enrichr, terms are ranked by their significance of overlap with the input gene set. These enrichment results can be used to suggest pathways, ontological terms, diseases, and drugs that may be associated with the input lncRNA.

The lncHUB2 lncRNA report pages also provide predicted biological functions for each lncRNA using an alternative method. These predictions are made by calculating the mean PCC between the lncRNA and the genes within each set of a gene-set library. *P*-values are computed to account for differences in set sizes. Terms in each gene-set library are ranked by the right- and left-tailed *P*-values to prioritize terms that have significant positive and negative correlations with the input lncRNA. Predictions are made with the following gene-set libraries from Enrichr: Mouse Genome Informatics (MGI) Mammalian Phenotypes (45), Gene Ontology Biological Processes (GO BP) (46), Kyoto Encyclopedia of Genes and Genome (KEGG) pathways (47), DisGeNET diseases (48) and transcription factors from ChEA (49) and ENCODE ChIP-seq (50). The prioritized terms are predicted functions that are likely associated with the lncRNA, and these are displayed as bar charts and can be downloaded as CSV files. Next, the lncHUB2 reports offer information about the relative expression of the queried lncRNA across 280 unique tissues and cell types, and 57 unique cell lines, in humans; and 27 unique tissues and cell types, and 20 unique cell lines, in mice. RNA-seq samples from ARCHS4 (38) were first automatically labeled by tissue and cell line, and then the expression statistics for each lncRNA were computed for each tissue, cell type, and cell line. These results are displayed as box plot graphs and can also be downloaded as CSV files.

**Figure 2.**
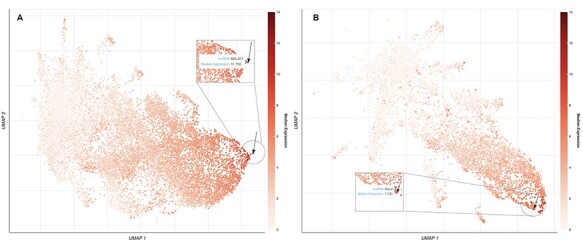
UMAP plots of 18 705 human lncRNAs and 11 274 mouse lncRNAs. (A) The lncRNAs level of intensity is by their median expression in the testis, where MALAT1 has the highest relative expression across tissues. The arrow is pointing to the location of MALAT1 on the UMAP plot. (B) lncRNAs level of intensity is by their log median expression in the peripheral nervous system, where Dleu2 has the highest relative expression across tissues. The arrow is pointing to the location of Dleu2 on the UMAP plot.

lncHUB2 also provides global visualizations of expression similarities for the collection of the 18 705 human lncRNAs and the 11 274 mouse lncRNAs in tissues, cell types, and cell lines. The uniform manifold approximation and projection (UMAP) (51) method is used for dimensionality reduction. It was applied to randomly selected RNA-seq samples from ARCHS4 (38). Within these visualizations, each dot represents a lncRNA and the proximity of each dot to other dots approximates the similarity between the lncRNAs expression vectors. A black arrow is pointing to the location of the queried lncRNAs. lncRNAs are colored based on their median expression in the tissue, cell type, and cell line where the queried lncRNA has the highest relative expression. An interactive version of this plot is available only in the lncHUB2 Appyter reports, where the user can select points on the plot by median expression across all tissues, cell types, and cell lines (Figure 2). Finally, lncHUB2 reports rank lists of small molecules and CRISPR KOs of single genes that are predicted to up- or downregulate the expression of the target lncRNA based on the LINCS L1000 data (35). A small molecule or single-gene KO perturbation is predicted to up- or downregulate an lncRNA if the corresponding L1000 up/down gene expression signature has a high mean PCC with the input lncRNA. Since the L1000 up and down gene signatures are of similar length, small molecules are ranked by mean PCCs; however, right-tailed *P*-values are also provided. The resultant tables are displayed within the lncRNA report and can be downloaded as CSV files.

### Benchmarking the lncHUB2 functional predictions

**Figure 3.**
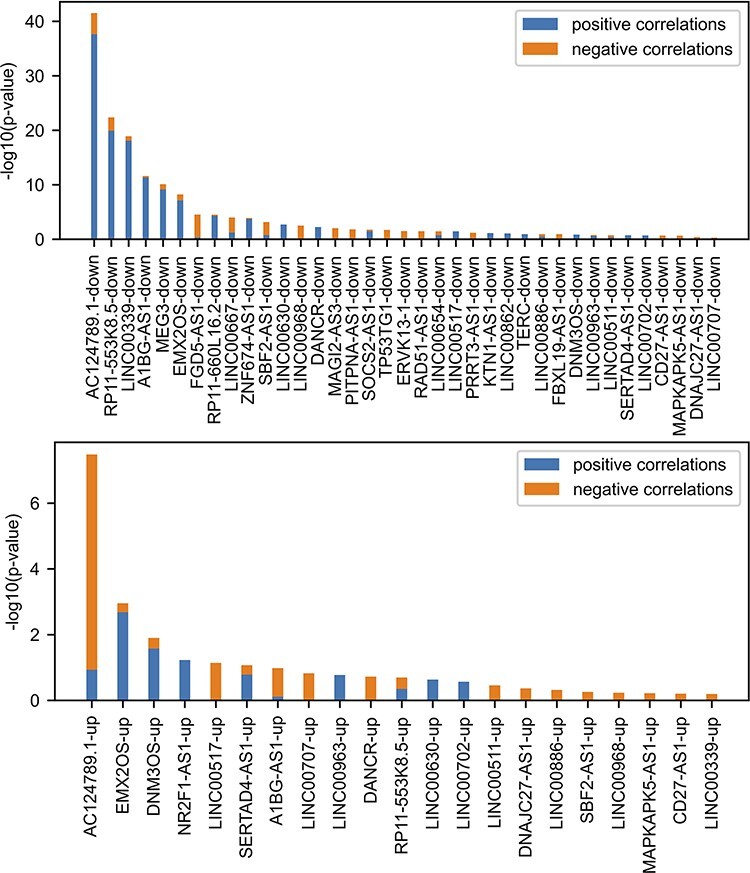
Comparing FANTOM6 lncRNA knockdowns followed by expression with gene–gene co-expression correlation data from ARCHS4. For each lncRNA in FANTOM6, we computed the significance of the overlap between the top 200 DEGs for each lncRNA knockdown (|log2 fold change (FC)| > 0.5; false discovery rate (FDR) < 0.05; |Zscore| > 1.645) in at least one knock-down condition and the top 200 most positively and top 200 most negatively correlated genes from the ARCHS4 gene–gene co-expression matrix using Fisher’s exact test. The *P*-values were then converted to −log10(*P*-values) and are visualized as stacked bar charts where the bottom part of the bar denotes the significance of overlap with positively correlated genes and the top part of each bar denotes the significance of overlap with the negatively correlated genes for each lncRNA. Only the top 37 lncRNA’s down genes with the most overlap and the top 21 lncRNA’s up genes with the most overlap are shown out of a total of 87 assessed.

lncHUB2 predicts biological functions, small molecules and single-gene perturbations that may modulate the expression of lncRNAs by leveraging gene–gene correlations generated from processed RNA-seq data. To benchmark the ability of this gene–gene correlation matrix to recover relevant biological functions, we utilize various gene-set libraries from Enrichr (52), data from lncRNA knock-down followed by expression, and lncRNA literature–based databases and publications. Up and down gene sets from lncRNA knock-down followed by expression (*n* = 99) were sources from FANTOM6 (53). The significance of the overlap between gene sets from FANTOM6 with lncRNAs most correlated genes contained within lncHUB2 was assessed using the Fisher’s exact test (Figure 3). Only the lncRNAs with the most overlap are shown. Several positively and negatively correlated genes with the same lncRNAs show significant overlap. Interestingly, the genes that are downregulated when the lncRNA is knocked down, and are positively correlated with the lncRNA, showed the most overlap (Figures S2 and S3). Hence, this suggests that these lncRNAs may act as positive regulators of transcription, but this could also be an indirect effect. Such relationship is most significant for the lncRNA AC124789.1, an lncRNA with no associated publications. Overall, we observe that gene–gene correlations could recover some of the same genes that are experimentally observed to be up- or down-regulated following lncRNA KOs.

Since it is known that lncRNAs are near the genes that they regulate, next, we aimed to examine how many of the most positively correlated genes with lncRNAs are *cis* or *trans*. To answer this question, we computed the portion of *cis* and *trans* genes mostly correlated between lncRNAs and coding genes, coding genes and coding genes, and non-coding genes and other lncRNAs for both humans and mice (*n* = 100) (Table 2). The ratio of *cis*-to-*trans* genes did not differ greatly between the groups except for coding genes that had a lower percentage of *cis* genes in both mice and humans. Thus, it does not appear that *cis* or *trans* genes are significantly more highly prioritized based on their co-expression, and most regulatory relations predicted via co-expression are *trans* for lncRNAs and coding genes.

**Table 2.** The percentage of the top 100 most correlated genes (human *n* = 62 548 and mouse *n* = 54 454), non-coding genes (human *n* = 42 278 and mouse *n* = 31 568), coding genes (human *n* = 20 270 and mouse *n* = 21 886) and lncRNAs (human *n* = 18 705 and mouse *n* = 11 274) with other lncRNAs.

		All genes	Coding genes- lncRNAs	Coding genes-coding genes	lncRNAs-lncRN
Mouse	*cis*	8.107% ± 9.814	8.087% ± 9.254	5.998% ± 10.074	8.725% ± 17.851
	*trans*	91.894% ± 9.814	91.913% ± 9.254	94.002% ± 10.074	91.275% ± 17.851
Human	*cis*	3.568% ± 2.587	3.637% ± 2.403	1.518% ± 3.817	4.366% ± 2.669
	*trans*	96.432% ± 2.587	96.363% ± 2.403	98.481% ± 3.817	95.634% ± 2.669

**Figure 4.**
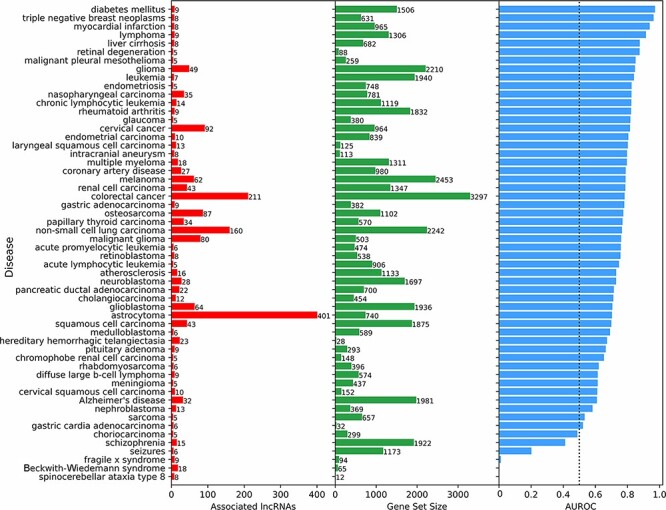
Predicting and evaluating the predictions of lncRNA–disease associations using gene–gene co-expression correlations. For each disease term from the DisGeNET gene-set library downloaded from Enrichr, the 18 705 human lncRNAs were ranked by their negative mean PCC with the corresponding gene set (bars at the center). The AUROC was calculated (bars at the right side of the plot) using the ranks of lncRNAs known to be associated with the same disease based on experimentally validated lncRNA–disease associations from LncRNADisease v2.0 (bars at the left side of the plot).

Next, we aimed to benchmark the prediction of lncRNA–disease associations. To achieve such benchmark, we compared the lncRNA–disease predictions based on co-expression with associations reported in the LncRNADisease database (18). Specifically, disease terms with at least five experimentally validated lncRNA associations from LncRNADisease v2.0 were used as a ‘gold’ standard. Disease terms from LncRNADisease v2.0 were mapped to the closest related disease term in the DisGeNET (48) gene-set library from Enrichr (52). For each disease term, the 18 705 human lncRNAs were ranked based on their mean PCC with the corresponding gene set from DisGeNET, and an area under the receiver operating characteristic (AUROC) curve was calculated to evaluate the ranking performance. For most diseases, prioritizing lncRNAs using mean co-expression performed much better than random (Figure 4). To confirm that this method was not prioritizing lncRNAs based on their expression levels alone, the rank and median expression for each lncRNA were examined (Figure S3). For many diseases, it seems that lowly expressed lncRNAs are prioritized, but this is not always the case. For example, spinocerebellar ataxia type 9 and Beckwith–Wiedemann syndrome have prioritized highly expressed lncRNAs associated with their known genes.

### Reporting and predicting lncRNA subcellular localization

**Figure 5.**
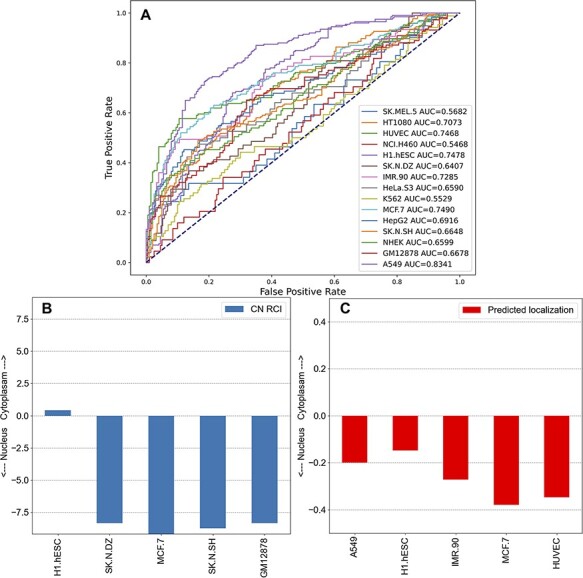
Unsupervised learning to predict the localization of lncRNAs by cell line. (A) Co-expression gene–gene correlations were used to predict localization values for each human lncRNA for the 15 cell lines in lncAtlas. For each human lncRNA, the 35 371 genes present across the cell types in lncAtlas were ranked by PCCs and ranks were multiplied by the existing RCIs from lncAtlas and summed. True positives and false positives were calculated for CN RCIs >1 and <−1 per cell line. (B) Subcellular localization RCIs for XIST, which are available for the displayed cell lines from lncAtlas. (C) Predicted subcellular localization for TSIX, an antisense gene to XIST. Subcellular localization information for TSIX is not available in lncAtlas for the five cell lines. These cell lines have the highest AUROCs as reported in (A).

The subcellular localization of lncRNAs is important for their function. The reports produced for each lncRNA in lncHUB2 contain information about the lncRNA subcellular localization in human cell lines sourced from lncAtlas (54). Although lncAtlas provides this information for many lncRNAs, we sought to expand this coverage by using an unsupervised learning approach to extend the coverage for the 18 705 human lncRNAs in the lncHUB2 database. Predicted localization per cell line was computed utilizing the ranked gene–gene expression correlations for the subset of genes contained within lncAtlas, providing a prediction between −0.5 and 0.5, indicating whether the lncRNA is predicted to be localized to the nucleus or the cytoplasm. The receiver operating characteristic (ROC) curves for each cell line show that for some cell lines this method reliably predicts the measured localization (Figure 5A). Within the lncHUB2 database, the predicted localizations are only shown for lncRNAs not contained within the lncAtlas database, for those lncRNAs within lncAtlas, lncHUB2 provides the measured localization. The lncHUB2 reports provide visualizations of the known (Figure 5B) and predicted (Figure 5C) localizations for the top five cell lines ranked by their respective ROC curves.

The ability to predict the subcellular localization varied per cell line. For many of the cell lines, the AUROC was <0.7, indicating that these predictions are unreliable. There are a few reasons that the ability to predict subcellular localization was inconsistent across the cell lines. First, the number of genes with subcellular localization information varied significantly per cell line from 5000 to 21 000. In general, the cell lines with the highest AUROCs were those with greater coverage of genes. For example, A549 (15 180 genes), HUVEC (15 145 genes), MCF7 (17 073 genes) and H1.hESC (21 382 genes) have relatively high AUROCs. Few cell lines with large gene coverage had relatively low AUROCs. For example, IMR90 contained information about 5599 genes and had an AUROC of 0.7285 and GM12878 contained information about 15 064 genes and had an AUROC of 0.6678. The inability of our approach to predict the subcellular localization for these two cell lines could be explained by lower correlations between the genes and lncRNAs in those cell lines. Since we want the users of lncHUB2 to only consider the most reliable predictions, we only report predicted subcellular localizations for the five best performing cell lines: A549, HUVEC, MCF.7, H1.hESC and IMR90.

### Case Study I: HOTAIR (ENSG00000228630)

To demonstrate the usability of lncHUB2, we first present a case study for a well-studied lncRNA called HOTAIR. HOTAIR was first discovered in 2007, where it was found to be located within the HOXC locus on Chromosome 12 and co-expressed with HOXC genes (55). It was initially shown that the 5ʹ end of HOTAIR interacts with the polycomb repressive complex 2 (PRC2) complex, while the 3ʹ end interacts with the LSD1/CoREST/REST repressive complex, and thus HOTAIR was theorized to serve as a scaffold for chromatin-modifying complexes (56). While HOTAIR’s interaction with PRC2 was theorized to play an essential role in PRC2-mediated transcriptional repression of the HOXD locus, more recent studies have disputed this finding. Instead, it was found that, independent of PRC2, HOTAIR overexpression led to small transcriptomic changes. Additionally, it was found that HOTAIR tethering to chromatin led to gene silencing and that PRC2 was dispensable in this process (57).

**Figure 6.**
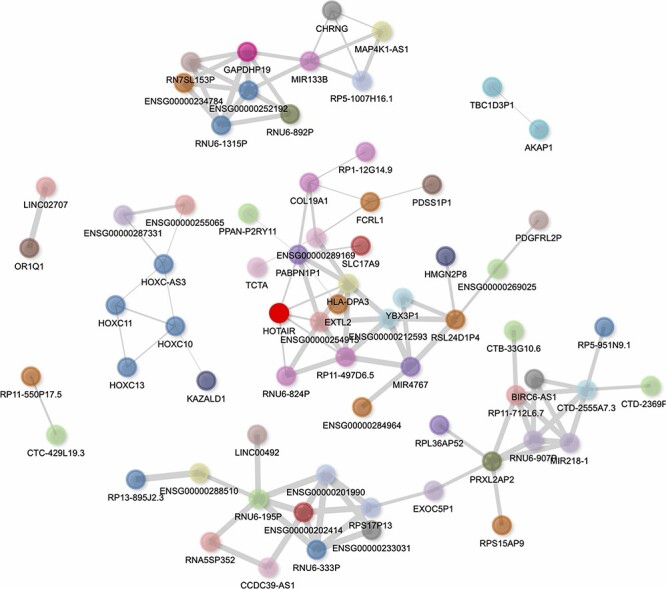
Interactive gene–gene co-expression network for the lncRNA HOTAIR. The HOTAIR gene–gene co-expression network contains the top 100 genes most correlated with HOTAIR. The thickness of the edges represents the magnitude of the PCCs, and nodes representing genes are colored by their chromosome of origin except for the queried lncRNA, which is colored in bright red. The network is pruned so that each node on average has less than three edges.

HOTAIR’s gene–gene correlation network can be visualized in the report generated by lncHUB2 for HOTAIR (Figure 6). In this network, the HOXC genes, including HOXC-AS3, HOXC10, HOXC11 and HOXC13, are visualized to the left of HOTAIR, showing that lncHUB2 gene correlations can recover known co-expression relationships. Interestingly, HOTAIR is directly connected to genes that are *trans*, which can be assessed through the nodes’ varying colors. Although these directly connected genes are not associated with HOTAIR in the literature, they could be interesting targets to investigate in conjunction with HOTAIR. Although HOTAIR has poor sequence conservation, its secondary structure is relatively conserved in mammals, suggesting its involvement in similar biological functions across different species (58). The HOXD locus encodes for transcription factors essential for development, and the dysregulation of HOXD genes has been linked to skeletal deformities in mice (59–61). HOTAIR-KO mice were previously shown to display upregulation of many genes, including HOXD genes, and exhibit skeletal abnormalities during development (62). This finding, however, has been disputed in a more recent study, where HOTAIR was found to be dispensable in normal mouse development, only eliciting a subtle effect on the *cis* genes Hoxc11 and Hoxc12 (63). HOTAIR has also been associated with a wide range of cancers and has shown to have oncogenic properties when overexpressed (64, 65). The upregulation of HOTAIR in normal breast epithelial cells was shown to induce hallmarks of cancer such as increased proliferation, migration and tumor invasion *in vivo* (66). High expression of HOTAIR has also been associated with increased chemoresistance and lower survival rates in lung cancer patients (67). Additionally, HOTAIR has been linked to heart disease and heart defects. In humans, HOTAIR upregulation has been linked to congenital heart disease (68) and HOTAIR polymorphisms have been linked to coronary artery disease (69). HOTAIR is also downregulated in patients with end-stage heart failure, and this observation was subsequently confirmed in a mouse model (70). Studies have revealed other functions for HOTAIR including involvement in protein degradation, inflammation, DNA damage response and cell signaling (71). Overexpression of HOTAIR alongside knock-down of miR-211 led to higher monocyte expression of the cytokines interferon (IFN)-γ, interleukin (IL)-6, IL-17, tumor necrosis factor alpha (TNF-α), IL-1β and IL-6 R (72). HOTAIR knock-down also induces changes in NFκB target gene expression, particularly for macrophages (73). HOTAIR was proposed to modulate DNA damage response through the activation of NFκB (74). The lncHUB2-predicted biological functions for HOTAIR recover many of these recently established functions including HOTAIR’s involvement in cancer, cell cycle, DNA damage response and immune signaling (Figures S4 and S5).

Across all tissues and cell types, lncHUB2 reports suggest that HOTAIR has the highest relative expression in synovial tissue and sarcoma (Figure S6A). There is evidence that HOTAIR promotes the progression of synovial sarcoma (75). The third highest tissue expression reported by lncHUB2 for HOTAIR is in the cervix, and HOTAIR has been associated with cervical cancer progression (76). lncHUB2 also displays the cell lines with the highest relative expression of HOTAIR (Figure S6B). The cell line with the highest expression of HOTAIR is normal human dermal fibroblasts (NHDFs) (Figure S6B), a cell line derived from primary NHDFs. Overexpression of HOTAIR in systemic sclerosis dermal fibroblasts induces the transcription factor GLI2, leading to the pro-fibrotic phenotype (77). The cell line with the second highest expression of HOTAIR is PANC-1, an epithelial cell line isolated from pancreatic duct carcinoma. Consistent with this observation, HOTAIR has shown to be highly expressed in pancreatic cancer (75). The next cell line with the highest expression of HOTAIR is G401, an epithelial-like kidney cell derived from an infant rhabdoid tumor. It was reported that HOTAIR is highly expressed in atypical rhabdoid tumors (78). Overall, lncHUB2 prediction about HOTAIR’s biological functions are supported by literature, as well as producing predictions about additional HOTAIR’s roles in normal physiology and disease.

### Case Study II: LINC00941(ENSG00000235884)

LINC00941 is a relatively under-studied lncRNA with <30 publications mentioning it as of late 2022. Most of the publications that discuss LINC00941 are cancer-related. LINC00941 has been implicated in various hallmarks of cancer across a variety of cancer types and has been shown to be a potential useful prognostic biomarker. LINC00941 expression has been used to successfully predict the survival of patients with lung adenocarcinoma (LAD) (79, 80) and was identified as a biomarker for hypoxia, which is associated with reduced survival of LAD patients (81, 82). Genes highly correlated with LINC00941 in LAD were found to be enriched for PI3K/AKT signaling and focal adhesion (83). LINC00941 has also been implicated in non–small cell lung cancer, where it was found to promote angiogenesis and tumor progression by sponging miR-877-3p, which is a negative regulator of VEGFA (84). LINC00941 is upregulated in oral squamous cell carcinoma (OSCC) and was shown to induce epithelial-to-mesenchymal transition (EMT) *in vitro* by associating with the heterogeneous nuclear ribonucleoprotein K (hnRNPK) (85). Another study found that LINC00941 activates the Wnt/β-catenin signaling pathway in OSCC (86). LINC00941 expression has shown to be positively correlated with gastric cancer progression (87, 88). LINC00941-knock-down experiments reduced gastric cancer cell proliferation and migration *in vitro* as well as tumor growth in mice (89). In pancreatic cancer, LINC00941 has shown to activate the LIMK1/Cofilin-1 pathway, which enhances cell proliferation and migration by regulating the actin cytoskeleton (90). In pancreatic adenocarcinoma, LINC00941 was found to sponge miR-873-3p and upregulate the expression of ATXN2 (91). In pancreatic ductal adenocarcinoma, LINC00941 was shown to promote glycolysis via Hippo signaling pathway activation (92). LINC00941 is upregulated in colon cancer and was shown to sponge miR-205-5p, leading to increased expression of MYC (93). Another study found that LINC00941 binds to SMAD4, which prevents SMAD4 ubiquitination and degradation and ultimately leads to the activation of the transforming growth factor beta (TGF-β) signaling pathway and subsequent EMT (94). Upregulation of LINC00941 has also been observed in patients with hepatocellular carcinoma (HCC) (95) and has been implicated in HCC relapse (96). LINC00941 upregulation has also been observed in patients with other liver diseases such as chronic hepatitis B and cirrhosis (95). In papillary thyroid cancer, it was found that TGF-β induces the transcription of LINC00941, which upregulates CDH6, an oncogene that promotes metastasis and EMT by modulating cytoskeleton adhesions, which hinder autophagy (97). LINC00941 is found to be a prognostic biomarker for head and neck squamous cell carcinoma (HNSCC) (98). In esophageal squamous cell carcinoma, LINC00941 was shown to sponge miR-877-3p and subsequently upregulate PMEPA1 (99). In addition to cancer, there is evidence in the literature that LINC00941 may be involved in cell differentiation. One study found that LINC00941 plays a role in regulating the differentiation of keratinocytes (100). Another recent study found that upregulated LINC00941 is associated with idiopathic pulmonary fibrosis (IPF), which is an incurable and progressive disease characterized by lung scarring (101). In IPF, LINC00941 was found to promote the differentiation of fibroblasts as well as increase cell proliferation and migration. It was also identified that ATF3 transcription factor enhances LINC00941 expression. Additionally, LINC00941 was shown to promote glycolysis and laryngocarcinoma progression through the PI3K/AKT/mTOR signaling pathway and its upregulation of PKM (102).

lncHUB2 predicts several of the known biological processes discussed above based on significant positive correlations between LINC00941 aND genes associated these terms (Figure S7). For example, two of the top predicted KEGG pathways are cell cycle and glycolysis, which are consistent with the literature. Most of the predicted GO biological processes are novel and revolve around DNA replication, metabolite biosynthesis, and immune processes (Figure S8). Predicted diseases from DisGeNET include cancers as the top two terms including composite lymphoma and childhood acute megakaryoblast leukemia. Although these cancer types were not previously associated with LINC00941 in the literature, they do coincide with LINC00941’s association with cancer progression in general. LINC00941’s predicted and potential role in immune regulation could be a potential new direction for better elucidating its pro-metastatic role in various cancers.

### Case Study III: MEG3 (ENSG00000214548)

**Figure 7.**
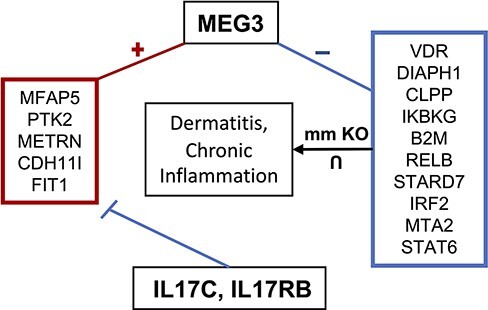
MEG3 involvement in dermatitis and chronic inflammatory response. MEG3 is co-expressed (positively correlated) with the genes contained in the left box, which are downregulated by IL17C and IL17RB. Genes in the right box represent the intersection of genes associated with dermatitis and chronic inflammation when knocked out in mice and which are negatively correlated with MEG3.

Maternally expressed gene 3 (MEG3) is another highly studied lncRNA shown to regulate cell proliferation and is considered a tumor suppressor (103). In cervical cancer cells, for instance, MEG3 is downregulated where it regulates the miR-21/PTEN axis, promoting cisplatin sensitivity (104). When knocked out, cervical cell proliferation and migration increase, while apoptosis is inhibited. Additionally, MEG3 is downregulated in multiple myeloma where it acts as an endogenous competitive RNA with miR-181a, inhibiting tumor progression and possibly regulating HOXA11 by sponging miR-171a (105). MEG3’s proposed tumor suppressor function has been theorized to act through both p53-dependent and independent pathways (106). MEG3 also functions to increase GluA1 subunits, a part of α-Amino-3-hydroxy-5-methyl-4-isoxazolepropionic acid (AMPA) receptors, on the plasma membrane, suggesting its function may be critical for long-term potentiation (LTP) (107). MEG3 has also been suggested to play a role in Parkinson’s disease where its expression is downregulated, acting as a biomarker for cognitive decline and disease stage (108). MEG3 has also been linked to both pro-inflammatory and anti-inflammatory mechanisms. For instance, in acute lung injury in a mouse model, MEG3 showed a protective effect on excessive inflammation through regulation of the TLR4/MyD88/NFκB pathway mediated by miR-93 (109). Another study found that MEG3 regulates the immune response to bacterial infection in lungs through binding to miR-138 competitively with IL-1β, increasing IL-1β concentration (110). lncHUB2 predicts that IL17RB and IL17C downregulate the expression of MEG3 based on the LINCS L1000 CRISPR-KO data (Table 3). MEG3 is co-expressed with anti-inflammatory genes that are downregulated by IL17C and IL17RB, and it is negatively correlated with genes that when knocked out in mice, it induces chronic inflammation and dermatitis (Figure 7). The top five predicted drugs that might upregulate MEG3 (Table 4) include RN-1734, a TRPV4 antagonist that was shown to reduce demyelination in central nervous system diseases as well as inhibit glial activation and IL-β and TNF-α production (111). Thus, RN-1734 may modulate excessive inflammatory responses through its predicted effect on MEG3 expression. lncHUB2 was able to predict many of the functions found in the literature for MEG3 such as reduced LTP and abnormal AMPA-mediated synaptic currents (Figure S9). In the left-tailed predictions, MEG3’s interaction with p53 was also recovered in the GO BP and KEGG libraries as well as its involvement with the NFκB pathway. Interestingly, DisGeNET top three predicted diseases were related to bacterial infection (Figure S10). Furthermore, many of the predictions from MGI, KEGG and GO BP were related to the dysfunction of synapses and neurotransmitter transport, supporting the role of MEG3 in LTP and Parkinson’s disease. MEG3’s role in regulating inflammatory response and neuronal functions warrants further exploration, especially considering its potential as a therapeutic target.

**Table 3.** Top five L1000 small molecules predicted to downregulate the expression of MEG3

Rank	CRISPR KO	Up/down	Mean PCC	*P*-value
1	PIGW	Down	0.0353754	3.74E−22
2	TUBD1	Down	0.04438307	1.61E−18
3	AJUBA	Down	0.03274345	6.60E−18
4	IL17RB	Down	0.05699057	1.94E−17
5	IL17C	Down	0.03780365	2.14E−17

L1000 small molecules are ranked by *P*-value between MEG3 and the genes in the up gene-set small-molecule signatures.

**Table 4.** Top five L1000 small molecules predicted to upregulate the expression of MEG3

Rank	Drug	Up/down	Mean PCC	*P*-value
1	RN-1734	Up	0.03492403	4.90E−19
2	Amprenavir	Up	0.02721223	2.67E−16
3	SA-441350	Up	0.03891477	1.22E−15
4	GW-788388	Up	0.03600107	3.35E−15
5	GALR3	Up	0.03087975	2.41E−14

L1000 small molecules are ranked by *P*-value between MEG3 and the genes in the up gene-set small-molecule signatures.

### Case Study IV: XIST (ENSG00000229807)

X-inactive-specific transcript (XIST) is one of the first lncRNAs that were discovered (112, 113). XIST is essential for X chromosome inactivation, and it is only expressed in female tissues (114). Recently, somatic activation of XIST was identified in a subset of male cancers (115). In ovarian cancer, XIST was shown to regulate the miR-506-3p/FOXP1 axis, in turn regulating autophagy and carboplatin resistance (116). lncHUB2 predicts terms that align with the known XIST’s functions. For example, the top term from MGI Mammalian Phenotypes was chromosomal instability (MP:0008866), and for GO BP, mitotic DNA replication initiation (GO:1902975) and nuclear cell cycle DNA replication initiation (GO:1902315) (Figure S11). Many of the other right-tailed predictions revolve around DNA regulatory mechanisms, suggesting a central role for XIST in such biological processes. XIST has strong measured nuclear subcellular localization (Figure 5B). For those lncRNAs that do not have predicted subcellular localization, lncHUB2 provides predicted localization. TSIX, an antisense lncRNA to XIST, is also predicted to be nuclear by lncHUB2. In mice, Tsix was shown to regulate Xist in *cis*, supporting this localization prediction (117).

### Case Study V: SAMMSON (ENSG00000245248)

Survival-associated mitochondrial melanoma-specific oncogenic non-coding (SAMMSON) is an lncRNA implicated in a myriad of regulatory mechanisms in a diverse set of cancers. For instance, SAMMSON is co-induced with the melanoma-specific oncogene MITF, and it is highly expressed in most melanomas, while knock-down of SAMMSON decreases melanoma cell viability and sensitizes melanoma to therapeutics (118). Additionally, the overexpression of SAMMSON in triple-negative breast cancer promoted cell proliferation, while the overexpression of p53 lessened this effect (119). In uveal melanoma, SAMMSON inhibition leads to the impairment of protein translation and mitochondrial function (120). In OSCC, SAMMSON expression was found to be elevated and correlated with OSCC stage, suggesting it may play an important role in this type of cancer (121). SAMMSON expression is also closely related to survival time and clinical stage in gastric cancer (122). SAMMSON knock-down was also observed to inactivate the PI3K/AKT pathway, suppressing the malignancy of glioblastomas (123).

lncHUB2 predicts a few of the functions documented in the literature in the left-tailed *P*-value results alongside many novel predictions (Figure S14). Many of the GO BP predictions for SAMMSON by lncHUB2 are related to apoptosis. This is consistent with its involvement with the mitochondria. Additionally, many of the predictions from right-tailed *P*-values encompass biosynthetic processes and metabolism (Figure S13). Since SAMMSON interacts with p32, which regulates mitochondrial homeostasis and metabolism, these predictions could elucidate additional functions for SAMMSON.

### Case Study VI: USP2-AS1 (ENSG00000240405)

USP2-AS1, also referred to as glycoLINC or gLINC, is an lncRNA that is known to form a scaffold to bring together several metabolic enzymes from the glycolysis pathway (124). Related to this role, USP2-AS1 is also implicated in cancer, and its overexpression may promote cancer growth. USP2-AS1 was observed to be a direct target of HIF1-α, and it is overexpressed in HNSCC, promoting cell proliferation and invasion through regulating DCAF13 activity (125). Additionally, USP2-AS1 was observed to be upregulated in ovarian cancer (126). It is a direct target of the transcription factor Myc, a key oncogene, promoting tumor progression through the regulation of E2F1 expression (127).

lncHUB2 predicts cellular senescence from the KEGG left-tailed *P*-value predictions and adenosine triphosphate (ATP) synthase complex assembly, both of which reflect findings in the literature (127) (Figure S16). Interestingly, its effect on glycolytic flux and glycolysis is not reflected in the predictions. The KEGG left-tailed predictions did, however, predict many associated cancers such as chronic and acute myeloid leukemia, bladder, prostate and thyroid cancers. DisGeNET left-tailed predictions also included a range of cancers such as neuroblastic tumors, giant cell glioblastoma and teratocarcinoma. Overall, lncHUB2 was able to identify that USP2-AS1 is associated with cancer progression. It is possible that such involvement is directly involved with enhancing glycolysis, but other possible mechanisms could be further explored.

## Discussion

lncHUB2 is a database, a website, and an Appyter that provides systematic knowledge about 18 705 human and 11 274 mouse lncRNAs. lncHUB2 contains existing knowledge and predictions about the biological functions and drug and disease associations for most of the known but under-studied human lncRNAs. Leveraging gene–gene co-expression correlations generated from publicly available RNA-seq data from thousands of independent studies, lncHUB2 can accurately predict the biological functions of lncRNAs, and prioritize >10 000 small molecules and >10 000 CRISPR-KO genes that would maximally up- or down-regulate the expression of each lncRNA. Overall, lncHUB2 is a significant upgrade of the original lncHUB web server. The initial version of lncHUB was using a similar approach to predict gene functions, but predictions were provided for only three libraries: KEGG, MGI and GO BP. In addition, predictions were made for only ∼4000 human lncRNAs. lncHUB2 has predictions made with more gene set libraries, predictions for the effects of small molecules, drugs, and CRISPR KOs on lncRNA expression levels, predictions and visulization of the lncRNAs secondary structure, publications about the lncRNA, expression of lncRNAs across tissues and cell lines, global visualization of the gene expression similarity between all human and mouse lncRNAs, and predictions about the cellular localization of lncRNAs within cell lines.

We present six detailed case studies that demonstrate how lncHUB2 can uncover and recover both new and previously known knowledge about lncRNAs. For the first case study, we selected HOTAIR, a well-studied lncRNA. lncHUB2 was able to recover HOTAIR’s association with HOXC genes , and through co-expression analysis, lncHUB2 pointed out previously established roles of HOTAIR involvement in cancer, cell cycle, DNA damage response and immune signaling. Additionally, lncHUB2 identified tissues where HOTAIR is highly expressed, especially in specific cancer types. For the second case study, we selected LINC00941, which is a relatively under-studied lncRNA with <30 related publications in PubMed. We found that lncHUB2 predicted some of the biological functions that have already been associated with LINC00941 such as cell cycle and glycolysis. Additionally, lncHUB2 predicted novel associations for LINC00941 such as involvement in regulating immune system functions and biosynthesis processes. MEG3, another highly studied lncRNA, had many of its known functions identified by lncHUB2 predictions such as reduced LTP and abnormal AMPA-mediated synaptic currents, as well as interactions with the p53 and NFκB pathways. Additionally, the predictions for L1000 small molecules and CRISPR-KO genes that may up- or down-regulate MEG3 expression provided a possible mechanism for how MEG3 could be involved in chronic inflammation and dermatitis. lncHUB2 was also able to recover the functions of XIST, an lncRNA responsible for X chromosome inactivation, predicting functions related to DNA initiation and replication. Additionally, for SAMMSON, an lncRNA that acts outside the nucleus, lncHUB2 provided predictions supported in the literature such as interaction with p53, apoptosis, and cell proliferation. Finally, for the lncRNA USP2-AS1, lncHUB2 predicted involvement in cellular senescence and ATP production , while the role of USP2-AS1 in glycolysis was not identified. lncHUB2 did, however, associate USP2-AS1 with a variety of cancers. Through these case studies we demonstrated how lncHUB2 can potentially predict biological functions for lncRNAs using both significant negatively and positively correlated annotated gene sets, and potentially modulating small molecules and CRISPR-KO genes. Overall, lncHUB2 has the potential to serve as a useful hypothesis generation tool for researchers studying lncRNAs.

Although lncHUB2 gene report pages provide reliable results, lncHUB2 has some limitations that should be discussed. For example, lncHUB2 makes predictions about lncRNA functions and disease associations by simply calculating the mean PCCs between a lncRNA and gene sets associated with biological functions, diseases, and small molecules. lncHUB2 predictions can potentially be improved by applying more complex machine learning algorithms. Recently, there has been an increase in applying machine learning methods to uncover knowledge about lncRNAs such as lncRNA–disease associations (128–132), lncRNA–protein interactions (133, 134), and lncRNA annotation (135). Another potential limitation of lncHUB2 is that it is using of a ‘global’ gene–gene co-expression matrix generated from randomly selected RNA-seq samples from ARCHS4 (38). Since gene co-expression can be context-specific, especially for genes that are variably expressed across different cell types and tissues, such as lncRNAs, selecting appropriate RNA-seq samples to produce a more accurate context-specific co-expression matrix can potentially improve predictions. These limitations can potentially be addressed by leveraging PrismEXP (136), an algorithm that automatically builds context-specific co-expression matrices and trains a regression model to improve unsupervised gene function predictions compared to using the global cross-tissue co-expression matrix. Observing significant positive or negative correlations does not indicate direct causality. Experimental evidence is likely needed for elucidating the regulatory mechanisms of each lncRNA. However, challenges remain with setting up such experiments because regulatory effects may be indirect (137). For example, despite a strong negative correlation observed after DNA damage between individual lncRNA/coding-gene pairs, namely, NOP14-AS1:NOP14 and LIPE-AS1:CEACAM1, direct causal effect could not be elucidated experimentally by perturbing these genes (138). This is just one example of how correlations may be the result of indirect system-wide effects, and the functional predictions produced by lncHUB2 should be viewed with caution and verified in experimental settings. The subcellular predictions provided in the lncHUB2 report also have some limitations. They are based on the same global gene–gene correlations and thus are affected by the same caveats hindering the functional predictions. Additionally, the composition and number of genes with subcellular localization values that are available for each cell line from lncAtlas varies. This makes our ability to robustly predict the localization of lncRNAs across cell lines uneven. Although we only report predictions for the top five performing cell lines, attention should be paid to the consensus of localizations across these cell-lines. Additionally, the magnitude of these predictions (closer to −0.05 or 0.5) reflects the strength of the prediction for that cell line and should be considered. More robust predictions across cell lines might be achieved with more complex machine learning models. Utilizing Deep Learning and other supervised learning approaches is one direction that warrants further exploration. Like the functional predictions produced by lncHUB2, localization predictions should also be viewed with caution and verified in the experimental setting.

## Conclusion

lncHUB2 is a database application and an Appyter that provides comprehensive knowledge about human lncRNAs, offering a wealth of information about 18 705 unique human and 11 274 mouse lncRNAs. By implementing lncHUB2 as an Appyter and as a simple web-based resource, we plan to routinely update the content within lncHUB2 without significant overhead. The comprehensive reports for each lncRNA in lncHUB2 include processed knowledge and prediction about the lncRNA’s secondary structure, the place of the lncRNA within gene–gene co-expression networks, predicted biological functions and pathways, disease associations, predictions about regulation by transcription factors, predictions about subcellular localization, measured expression levels across various tissues, cell types, and cell lines, and predictions about small molecules and single gene CRISPR-KOs that may modulate the lncRNA expression. Altogether, lncHUB2 is a useful resource for hypothesis generation, particularly for those lncRNAs whose functions have yet to be elucidated.

## Materials and methods

### Secondary structure predictions

Complementary DNA (cDNA) sequences for lncRNAs were downloaded from Ensembl (Homo_sapiens.GRCh38.ncrna.fa and Mus_musculus.GRCh38.ncrna.fa). Using the default settings, RNAfold v2.5.0 (41) was applied to the cDNA sequence of the canonical transcript for each lncRNA. Secondary structure predictions were not made if the cDNA sequence was not available or if the cDNA sequence exceeded the maximum length RNAfold can manage.

### Creating the gene–gene co-expression matrix

Separately for humans and mice, 6000 samples were randomly selected from the ARCHS4 bulk RNA-seq samples. The samples were separately aligned with kallisto (43) against GENCODE v41 and vM30, which corresponds to Ensembl 107 for both humans and mice. Genes with 0 reads across all 6000 samples were removed, resulting in 62 548 genes for humans and 53 454 genes for mice. Samples were then log2-transformed and quantile-normalized. Gene–gene correlations were calculated with PCCs. To avoid misleading high correlations between lowly expressed genes, pairwise gene–gene correlations were only calculated if at least one gene was expressed (normalized expression value >0) in ∼30% of the 6000 randomly selected samples. Otherwise, the pairwise correlation was set to 0.

### Gene mapping

Ensembl gene IDs from ARCHS4 RNA-seq samples were converted into gene symbols. First, lncRNAs were identified using an lncRNA annotation file downloaded from GENCODE (gencode.v41.long_noncoding_RNAs.gtf). Using the ‘gene_id’ and ‘gene_name’ columns, Ensembl IDs for annotated lncRNAs were converted to gene symbols. In total, 18 705 human and 11 274 mouse lncRNAs were identified in ARCHS4. This difference in the number of lncRNAs annotated in GENCODE v41 (19 095) compared to those included in lncHUB2 (18 705) is due to a filtering step in which genes with 0 reads across the 6000 randomly selected samples were removed when creating the gene–gene co-expression matrix used to make the predictions. Ensembl IDs not identified to be an lncRNA in GENCODE V41 were then converted to approved gene symbols using **Human Genome Organisation** (HUGO) Gene Nomenclature Committee (HGNC) with BioMart (139) (on 3 October 2022). Ensembl IDs that are mapped to the same symbol were manually checked and converted using the GeneCards database (140). Additionally, FANTOM-CAT (v6) (53) was used to label additional human lncRNAs. For genes without a gene symbol, the Ensembl ID was retained.

### Creating a gene–lncRNA network visualization

All pairwise correlations between the top 100 genes correlated with the input lncRNA are extracted. The three edges with the highest correlation per gene (node) are used to initialize the network. Edges with weights <0.3 are dropped. To further prune the network, the edges with the lowest weight for each hub node are dropped. At the start of the pruning process, a hub node is defined as a node with >10 edges. The pruning process is repeated until the network has an average of <3 edges per node. The top five edges for the input lncRNA are shown regardless of their weights.

### Benchmarking gene prioritization using co-expression

For benchmarking the gene prioritization using co-expression, the significance of overlap between the top 200 most positively correlated and top 200 most negatively correlated genes were computed with the observed DEGs from FANTOM6 (53). The Fisher’s exact test was used to assess this significance.

### Assigning *cis* and *trans* modes to lncRNAs

To compute *cis* and *trans* modes for each lncRNA, the chromosomal location for each lncRNA was first sourced from GENCODE v41 and vM30 as well as from Ensembl 107 through BioMart (139). Then, the top 100 correlated lncRNAs from the ARCHS4 (38) gene–gene co-expression correlation matrix were assigned *cis* or *trans* mode if they resided on the same chromosome (*cis*) or a different chromosome (*trans*). The reported statistics are an aggregation of the proportion of *cis* and *trans* modes for all lncRNAs.

### Predicting subcellular localization values using co-expression

An unsupervised learning approach was utilized to predict subcellular localization values for all the human lncRNAs contained within the lncHUB2 database. First, the gene coverage across lncAtlas cell types was aggregated and the subset of overlapping genes in the co-expression matrix was retained in addition to the human lncRNAs, resulting in 35 371 genes. For each lncRNA, the remaining genes were ranked by their PCC with the lncRNA. These ranks were scaled to values between 0 and 1. Thus, the more correlated a gene was to a given lncRNA, the closer its rank was to 1, and the less correlated a gene was to a given lncRNA, the closer its rank value was to 0. These rank values were multiplied by the cytoplasm/nucleus relative concentration index (CN RCI) from lncAtlas if such a value existed and summed to produce a single value for each lncRNA. This process was repeated for each lncRNA, and then scores across all lncRNAs were normalized between 0 and 1. ROC curves were then calculated for each cell line, utilizing RCIs >1 and <−1 provided from lncAtlas as true positives and false positives. To report values similar to the RCIs provided by lncAtlas, these scores were shifted to a range between −0.5 and 0.5. The top five performing cell lines were then selected, and these are reported for those lncRNAs that do not have localization values in lncAtlas.

### Extracting lncRNAs and GO biological processes from the literature

To collect PMIDs and dates for publications associated with lncRNAs, the PyMed Python library was utilized. The Ensembl ID, lncRNA gene symbol from GENCODE (40) and any symbols/previous symbols found in the HGNC database using BioMart (139), along with the terms ‘lncRNA’ or ‘long non-coding RNA’, were used to query PubMed (e.g. (ENSG00000228630 OR HOTAIR) AND (lncRNA OR long non-coding RNA)). All PMIDs and dates were extracted for each lncRNA. To collect PMIDs associated with GO biological processes, each term was submitted to PubMed using the PubMed API and the top 20 000 PMIDs were extracted.

### Calculating *P*-values

To assess the significance of an lncRNA’s mean PCC with a gene set of varying sizes, *P*-values were calculated with respect to each term in the gene set libraries. Each term therefore had its own unique mean and SD for calculating *z*-scores. The cumulative normal distribution was used to convert *z*-scores into right- and left-tailed *P*-values, which are then converted to −log10 of the *P*-value for visualization in the lncHUB2 report.

### Labeling ARCHS4 samples by tissue type and cell line

The sample descriptions from the ARCHS4 metadata were automatically searched for tissue and cell-line terminology. To create files with tissue and cell-line terminology, metadata files were first downloaded from CellMarker (141) and the Cancer Cell Line Encyclopedia (142). To generate a cell type to tissue mapping file, the ‘Human_Cell_Markers.txt’ file was downloaded from CellMarker (http://biocc.hrbmu.edu.cn/CellMarker/). The ‘tissueType’ and ‘cellName’ columns were used. This file was manually cleaned to remove duplicate cell-type to tissue mappings. Rare cell types were removed, and some tissues were renamed to their broader tissue type categories for simplification. For example, the ter endometrium was changed to uterus. This mapping file was further reduced by only keeping cell types that were present in the ARCHS4 sample descriptions. Finally, a few tissue types without cell-type mappings were manually added, for example the stomach, adrenal cortex, intestine, oral cavity, soft tissue, colorectal and gallbladder. All terms were standardized by removing symbols and converting to lowercase. ARCHS4 samples were then labeled by tissue type using a basic text search. First, sample descriptions were searched for cell types and then labeled with the corresponding tissue. If no cell type was detected, a basic text search was performed with tissue names. Samples that had multiple labels were manually labeled by reading the sample description. To create a list of cell lines for the text search, the ‘sample_info.csv’ metadata file was downloaded from the DepMap portal (143). The ‘stripped_cell_line_name’ column was used, and all cell lines were converted to lowercase. ARCHS4 samples not labeled with a tissue were searched for cell lines. Cell lines with three or less characters had the word ‘cell’ appended to them before the text search to prevent false-positive matches. The number of samples labeled with each tissue and cell is included in the downloadable CSV files for tissue or cell-line expression for each lncRNA in the field labeled ‘count’.

### Calculating tissue- and cell-line-specific lncRNA expression

In the ARCHS4 data, all samples that were labeled with a tissue type or a cell line were separately collected, and only counts for lncRNAs were kept. The expression statistics for each lncRNA was then calculated for each tissue and cell line. Tissues with <20 samples were removed before statistics were calculated for each lncRNA, except for mouse cell lines for which the threshold was set at 10, increasing the reported mouse cell lines to 20.

### LncRNA UMAP visualization for tissues and cell lines

A total of 3000 randomly selected ARCHS4 samples were log2-transformed and quantile-normalized. UMAP was applied to each dataset and then plotted with a scatter plot. Each lncRNA can be colored by median expression for each tissue type to visualize tissue-specific lncRNAs and lncRNA–lncRNA similarity.

### Prioritizing small molecules and CRISPR-KO genes that modulate lncRNAs

Processed into consensus signatures created from the L1000 characteristic direction (144), up- and downregulated gene sets for chemical perturbations (l1000_cp.gmt) and CRISPR-KO genes (l100_xpr.gmt) from SigCom LINCS (145) were downloaded from Enrichr (40). For each gene set, all 15 862 lncRNAs were ranked by mean PCC with the up/down genes from the GMT file. The top 1000 lncRNAs were retained for each up and down gene-set signature. A new GMT file was then created with each lncRNA as the ‘term’ and all small-molecule perturbations ranked by mean PCC as the ‘set’ members. This resulted in 13 043 lncRNAs with predicted small molecules and 12 899 lncRNAs with predicted CRISPR-KO genes in humans and 7951 lncRNAs with predicted small molecules and 7991 lncRNAs with predicted CRISPR-KO genes in mice. Small-molecule and CRISPR-KO gene predictions can then be separated based on the direction of the signature. If an lncRNA is associated with an ‘up’ small molecule or CRIPSR-KO gene set, this small molecule/gene is predicted to upregulate the lncRNA and vice versa.

### Appyter and web portal development

Initially, to gather knowledge about lncRNAs, lncHUB2 was implemented as a Jupyter Notebook workflow coded in Python. The notebook was converted into an Appyter (36). Appyters provide a rapid path to convert Jupyter Notebooks into full-stack web-based applications by inserting Jinja template code to specify user input form fields. Using the Appyter software development kit, a modified notebook is then compiled into a fully functional light-weight bioinformatics application. lncHUB2 is served on the Appyters Catalog. We ran the lncHUB2 Appyter with the input of the human and mouse lncRNAs, and the figures and files produced from the lncHUB2 Appyter were stored in a S3 bucket on Amazon Web Services. The lncHUB2 website is a Flask-based application that instantly displays the precomputed and stored figures, tables, and files produced by the lncHUB2 Appyter. The application and its dependencies run in a Docker virtual machine, which is served on a four-node cluster managed with Kubernetes. The front end of the application and its styling are implemented with JavaScript, Bootstrap, and HTML.

## Supplementary Material

baad009_Supp

## Data Availability

The gene-gene correlation matrix used to create the predictions for lncHUB2 is available for download from the ARCHS4 site at: https://maayanlab.cloud/archs4/download.html. All processed and inferred lncRNA data served on lncHUB2 is available for download from: https://maayanlab.cloud/lncHUB2 A database dump of the entire lncHUB2 database can be made available upon request from the authors.
