# Supplementary material for: lncHUB2: aggregated and inferred knowledge about human and mouse lncRNAs
Source: Database (Oxford). 2023 Mar 4;2023:baad009. doi: 10.1093/database/baad009 (PMC9985331; doi:10.1093/database/baad009)
Supplement: baad009_Supp [file baad009_supp.zip › suppl_data/Supplementary-file-11242022.pdf]

SUPPLEMENTARY FILE

for

lncHUB2: Aggregated and Inferred Knowledge about Human and Mouse lncRNAs

Giacomo Marino[1], Megan L. Wojciechowicz[1], Daniel J.B. Clarke[1], Maxim V. Kuleshov[1], Zhuorui Xie[1], Minji Jeon[1], Alexander Lachmann[1], Avi Ma'ayan[1,*]

[1]Department of Pharmacological Sciences, Department of Artificial Intelligence and Human Health, Mount Sinai Center for Bioinformatics, Icahn School of Medicine at Mount Sinai, One Gustave L. Levy Place, Box 1603, New York, NY 10029, USA

*To whom correspondence should be addressed: avi.maayan@mssm.edu

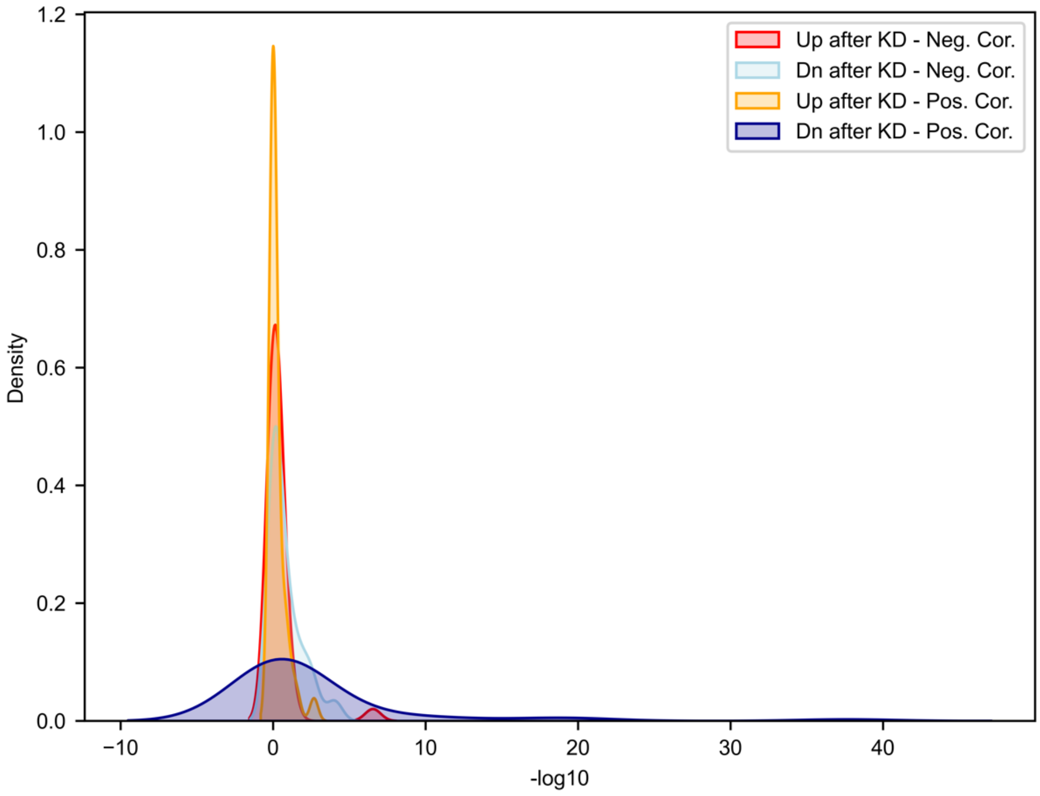

Fig S1. Pearson Correlation Coefficients and p-values for lncRNA-GO Biological Process co-mentions. Histograms display the distribution of mean PCCs, right-tailed, left-tailed, and two-tailed p-values for the top 200 co-mentions compared to 6,032 GO Biological Process terms for each of the top 10 published lncRNAs.

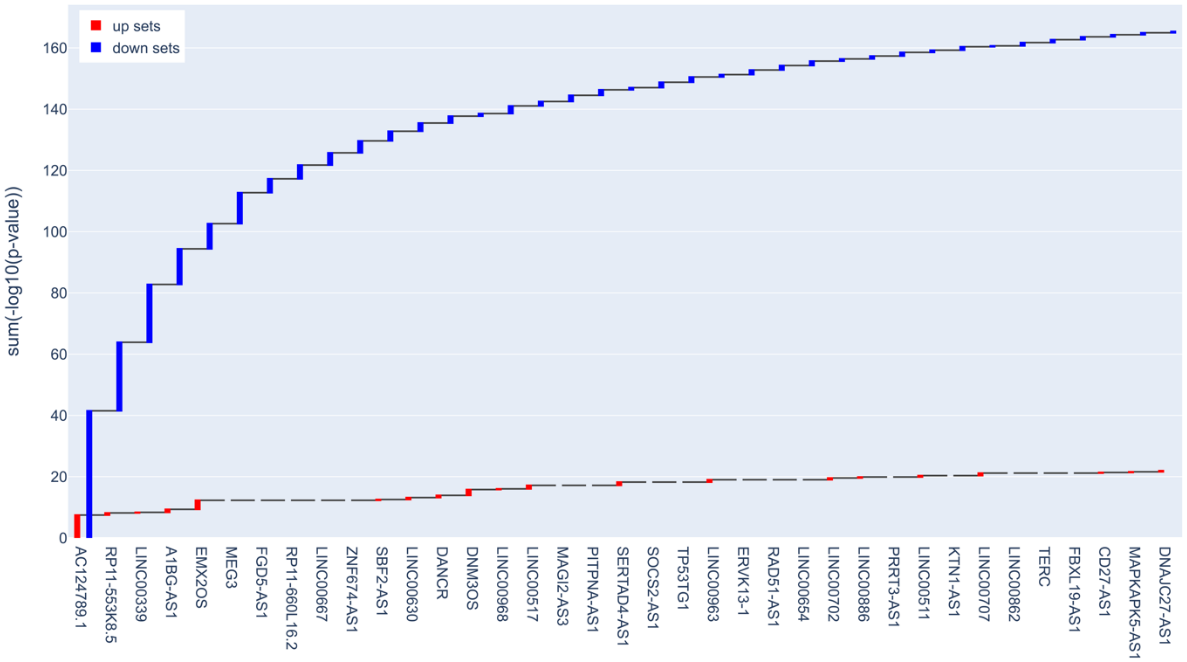

Fig S2. Overlap of the top 200 co-mentioned GO Biological Process between the most frequently studied lncRNAs. (A) The frequency of co-mentions for the top 200 co-mentioned GO terms for each lncRNA. **(B)** A heatmap displaying the number of overlapping co-mentioned GO terms for all pairwise lncRNAs. **(C)** Bar chart quantifying the degree of shared co-mentioned GO terms between all lncRNAs.

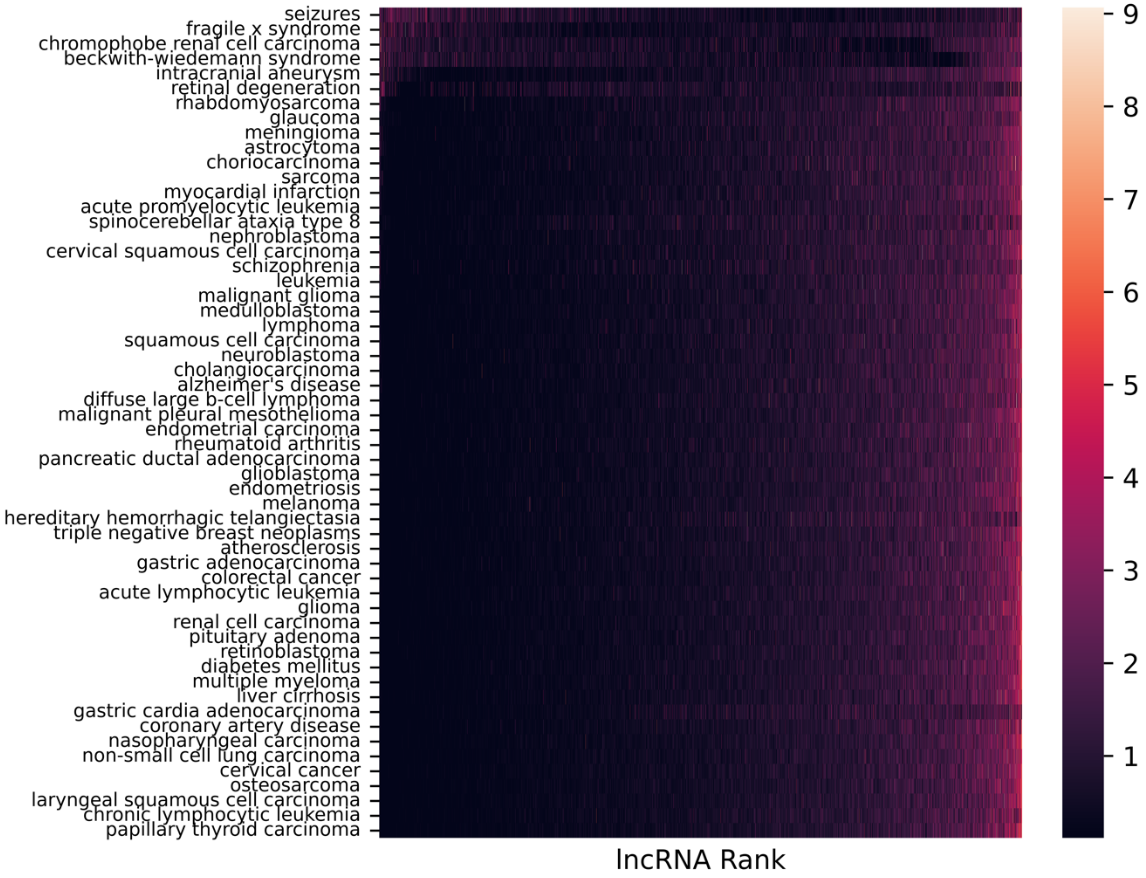

Figure S3. lncRNA expression values for predicted lncRNA-disease associations. 10,000 randomly selected samples from ARCHS4 were log2 and quantile normalized. The median expression for the 18,705 human lncRNAs was calculated. lncRNAs prioritized for each disease term are colored by their median expression.

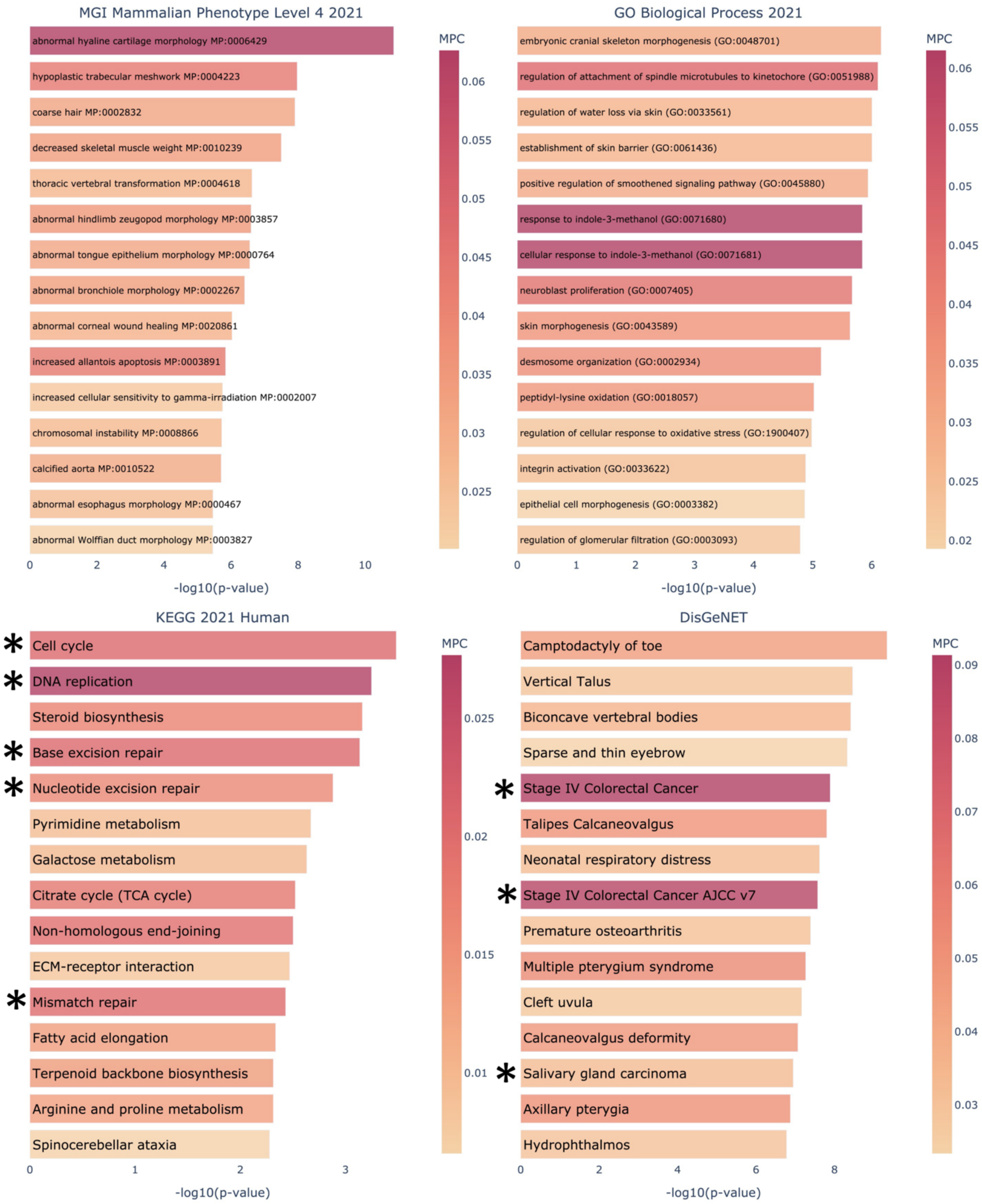

Figure S4. Predicted positively correlated biological functions for HOTAIR generated by lncHUB2. For each gene set library, biological terms are ranked by the right-tailed p-value for the mean PCCs between HOTAIR and each gene set. luncHUB2 displays the top 15 terms from each gene set library. Black asterisks represent functions that are associated with HOTAIR through a literature search.

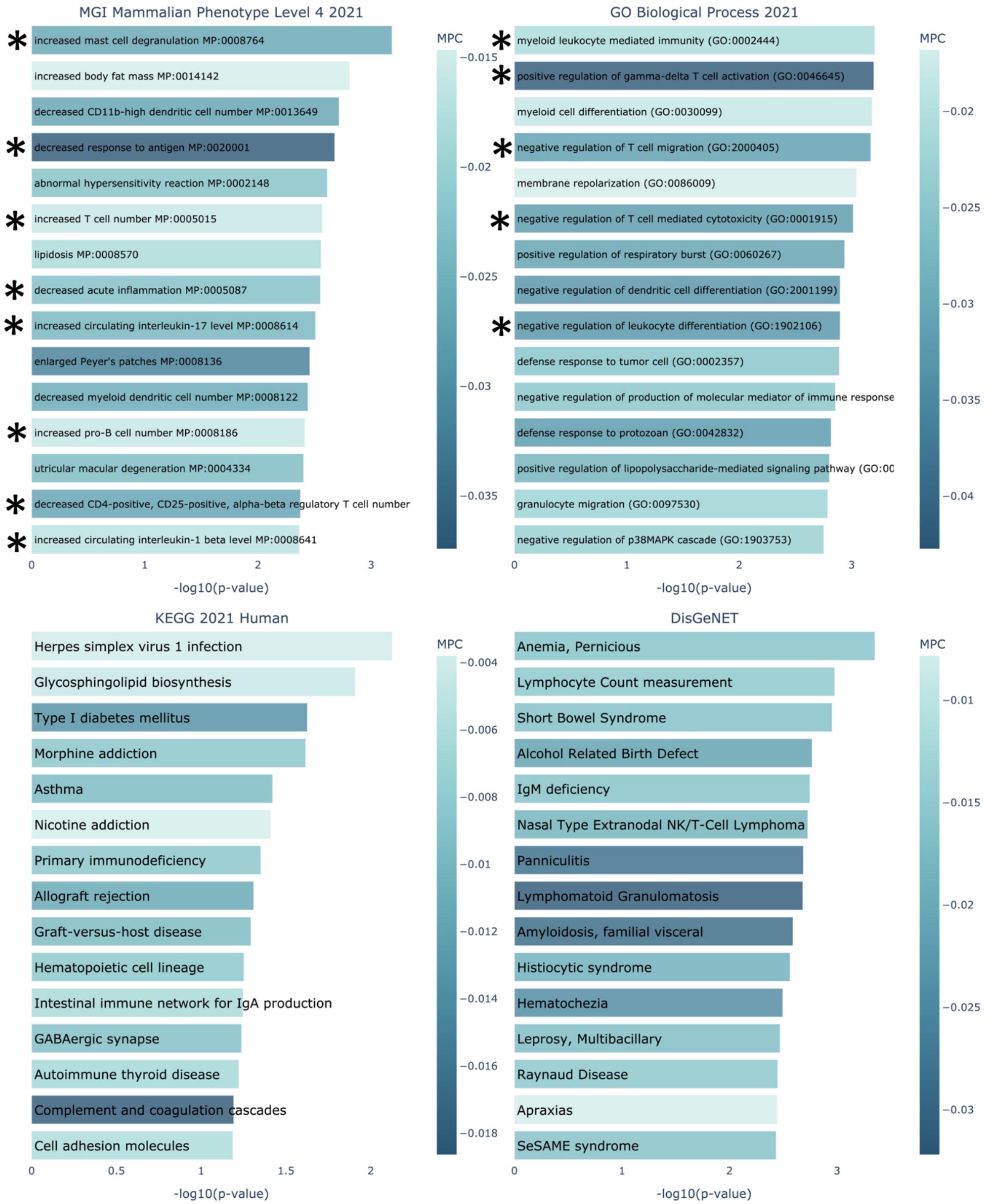

Figure S5. Predicted negatively correlated biological functions for HOTAIR generated by lncHUB2. For each gene set library, biological terms are ranked by the right-tailed p-value for the mean PCCs between HOTAIR and each gene set. luncHUB2 displays the top 15 terms from each gene set library. Black asterisks represent functions that are associated with HOTAIR through a literature search.

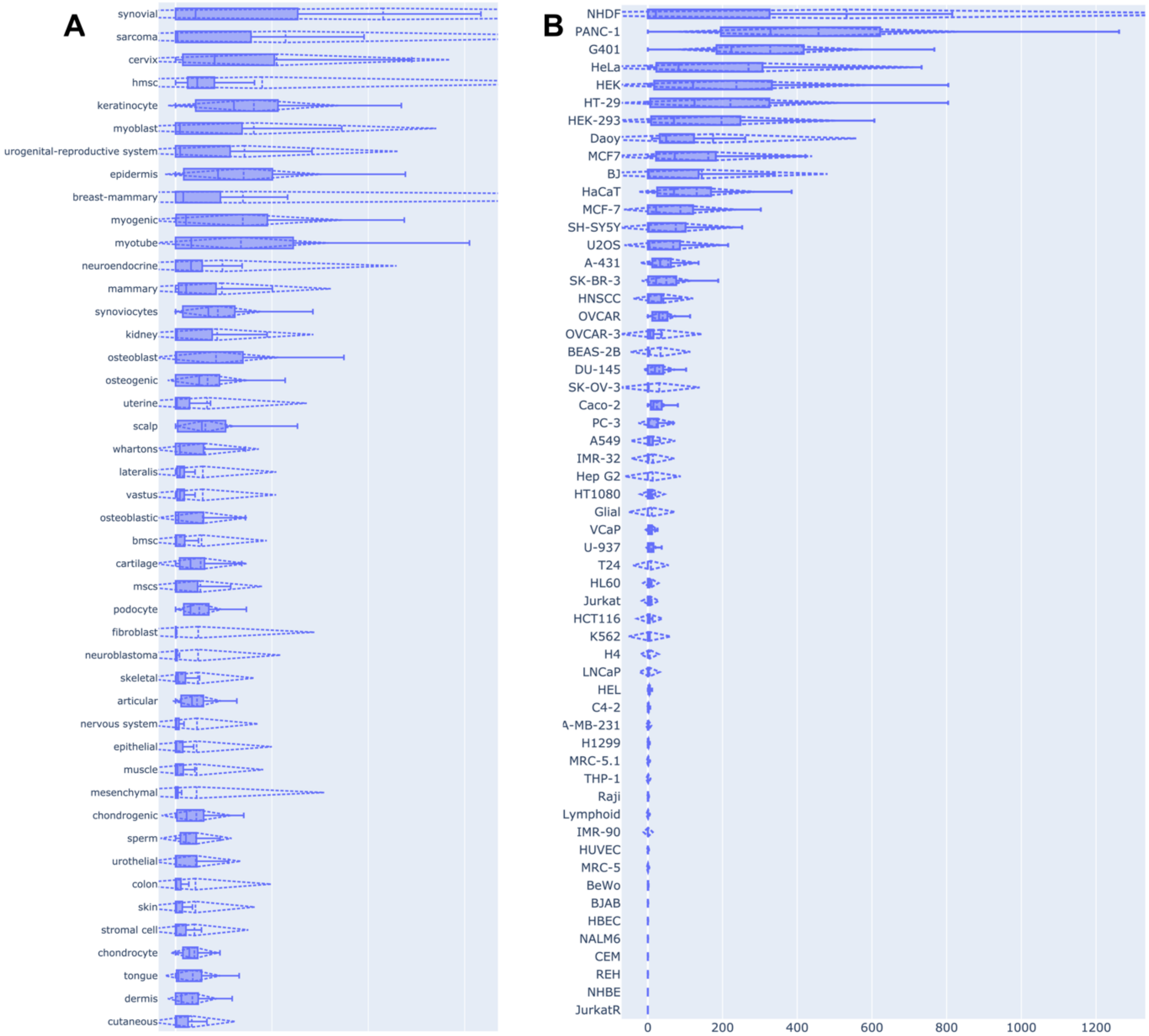

Figure S6. Expression statistics of HOTAIR across various tissues and cell lines. Expression statistics computed from ARCHS4 matrix with 280 annotated tissues and cell types and 57 annotated cell lines in humans.

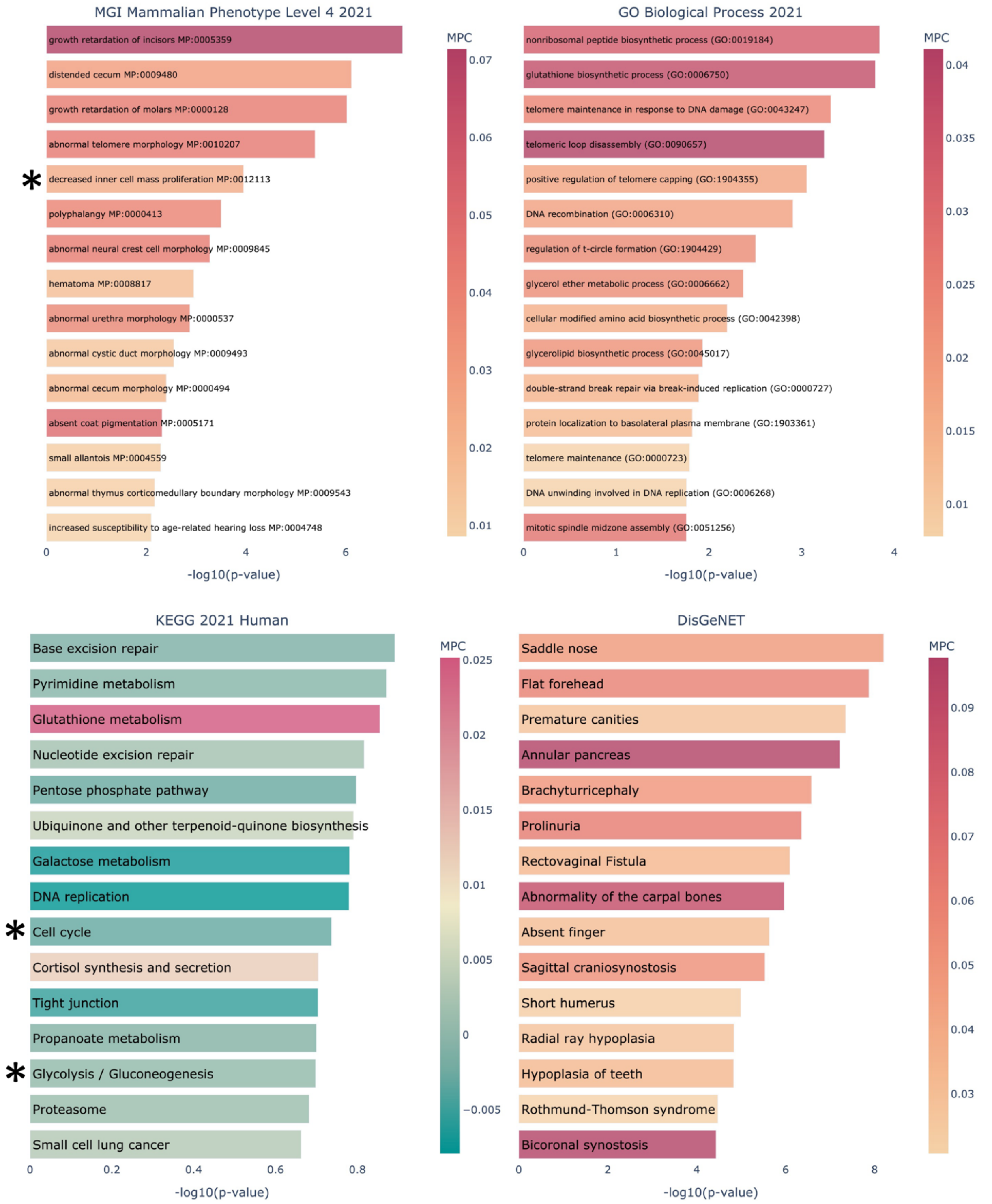

Figure S7. Predicted positively correlated biological functions for LINC00941 generated by lncHUB2. For each gene set library, biological terms are ranked by the right-tailed p-value computed for the mean PCC between LINC00941 and each gene set. luncHUB2 displays the top 15 terms from each gene set library. Black asterisks represent functions that are associated with LINC00941 through a literature search.

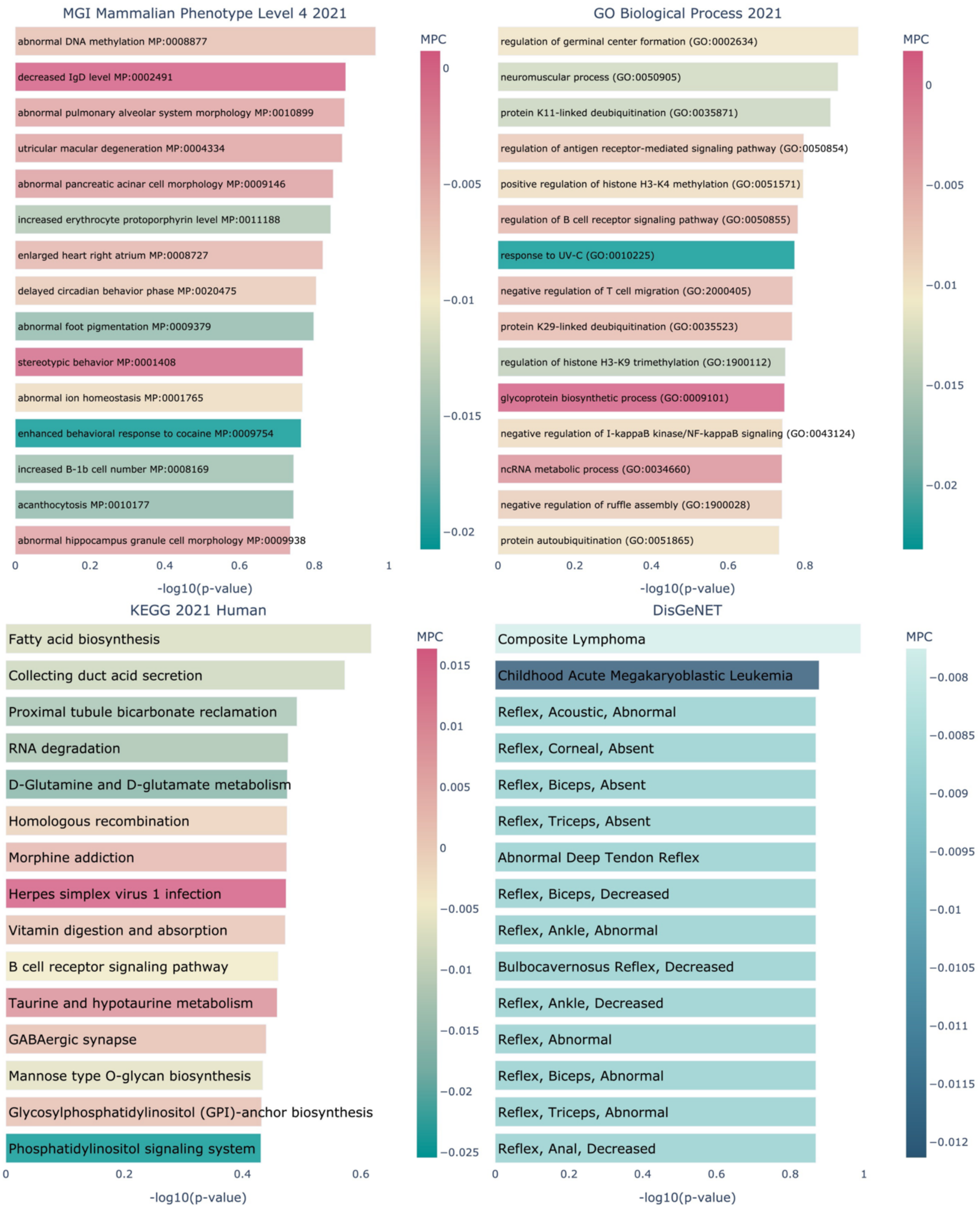

Figure S8. Predicted negatively correlated biological functions for LINC00941 generated by lncHUB2. For each gene set library, biological terms are ranked by the left-tailed p-value for the mean PCC between LINC00941 and each gene set. luncHUB2 displays the top 15 terms from each gene set library. Black asterisks represent functions that are associated with LINC00941 through a literature search.

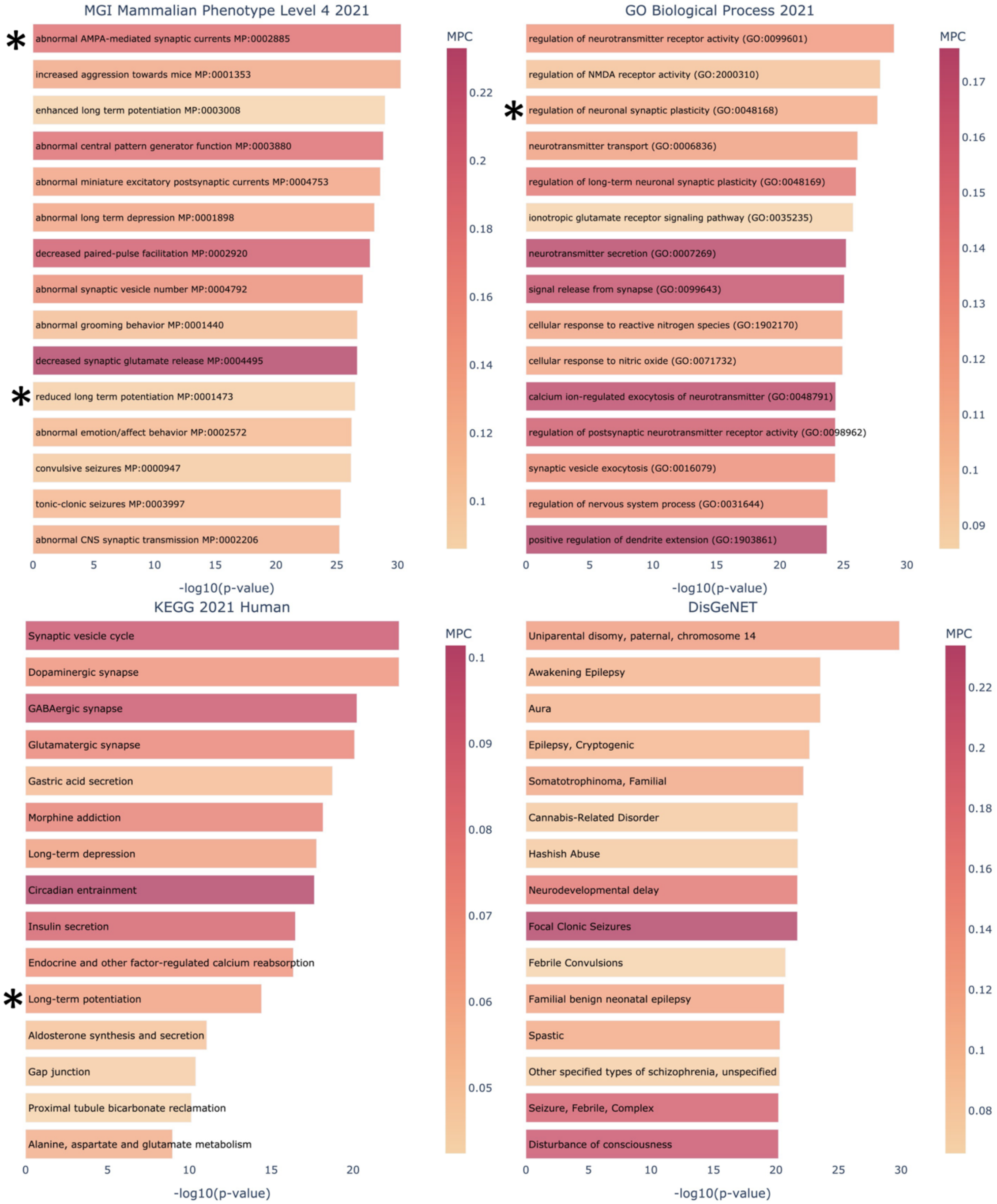

Figure S9. Predicted positively correlated biological functions for MEG3 generated by lncHUB2. For each gene set library, biological terms are ranked by the right-tailed p-value computed for the mean PCC between MEG3 and each gene set. luncHUB2 displays the top 15 terms from each gene set library. Black asterisks represent functions that are associated with MEG3 through a literature search.

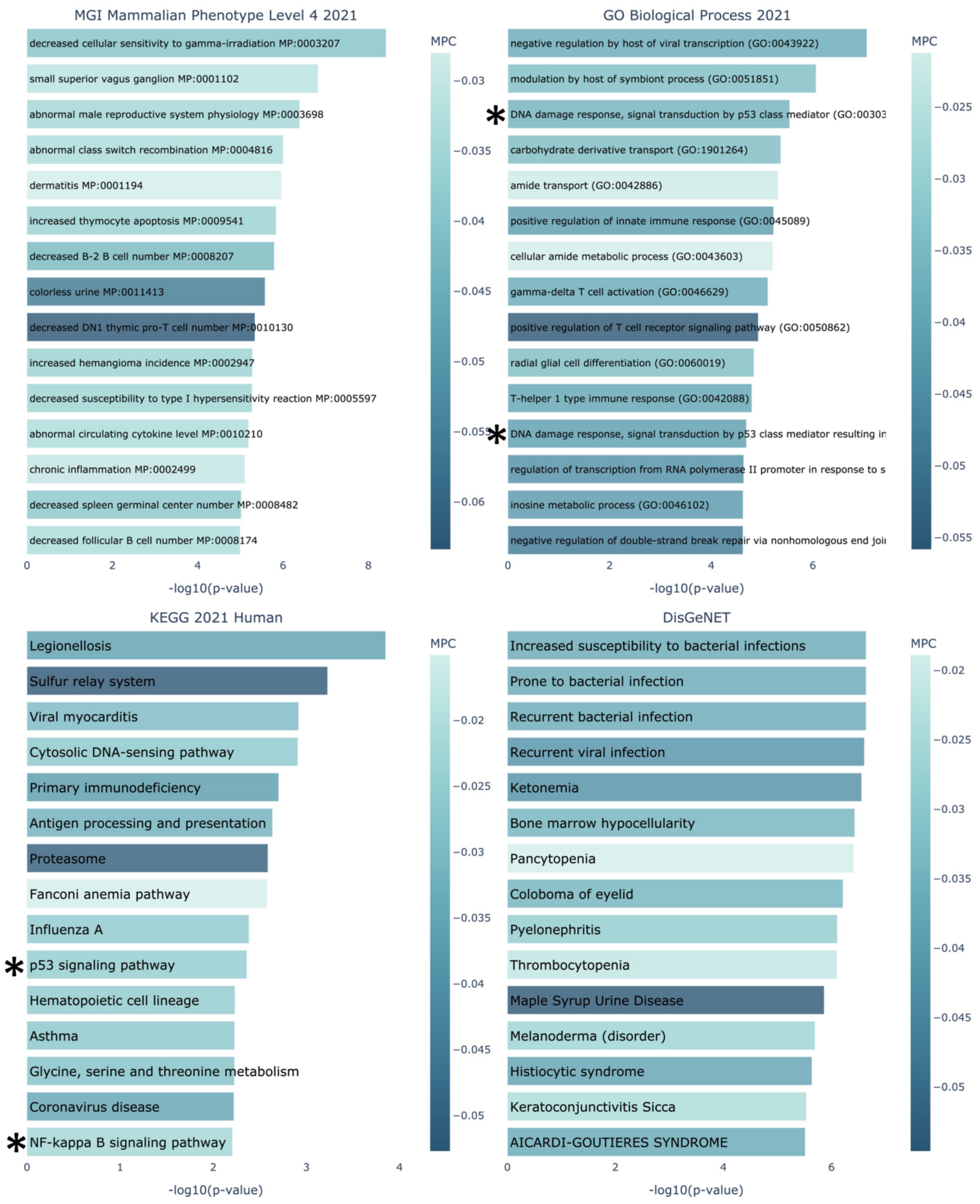

Figure S10. Predicted negatively correlated biological functions for MEG3 generated by lncHUB2. For each gene set library, biological terms are ranked by the left-tailed p-value for the mean PCC between MEG3 and each gene set. luncHUB2 displays the top 15 terms from each gene set library. Black asterisks represent functions that are associated with MEG3 through a literature search.

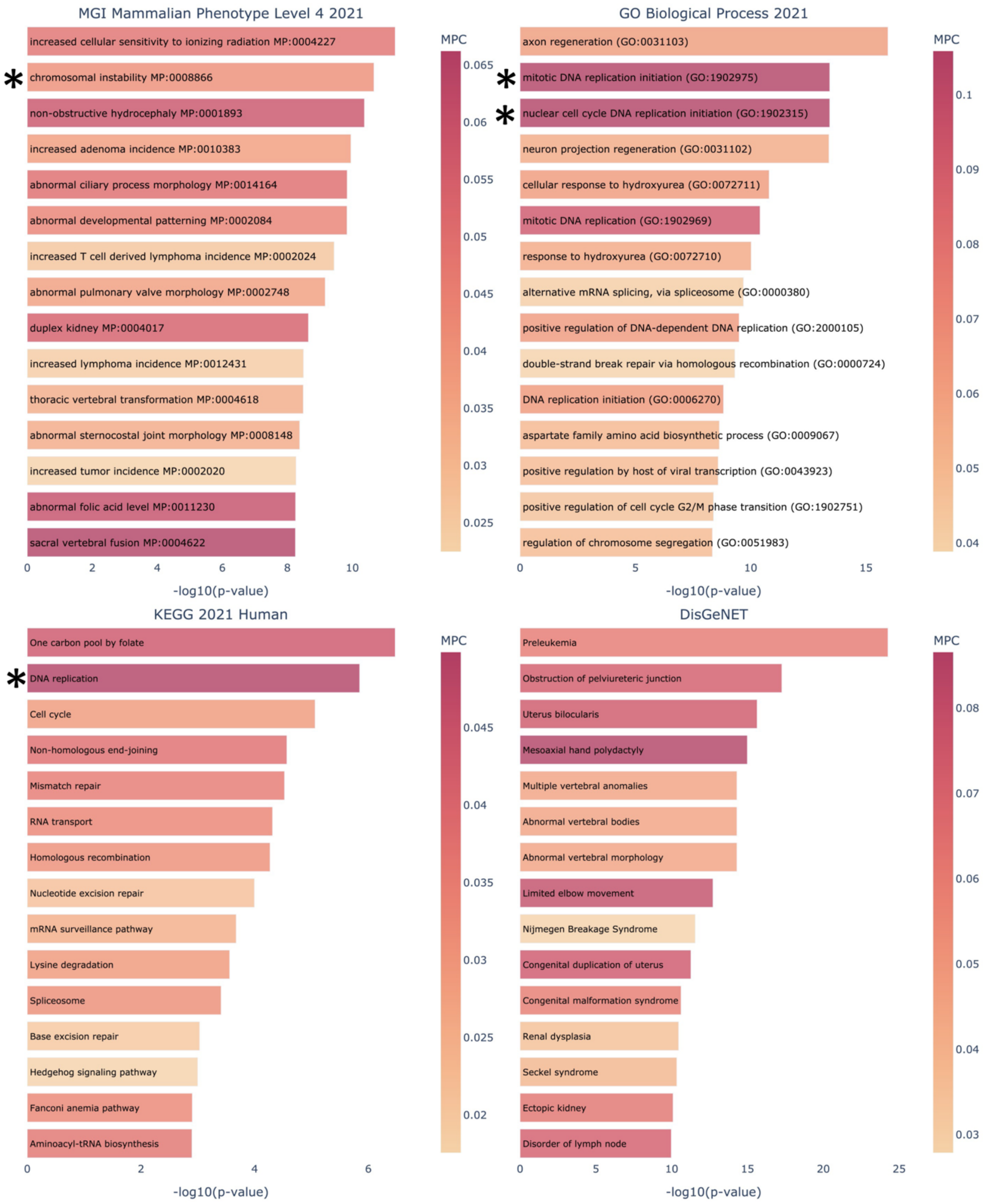

Figure S11. Predicted positively correlated biological functions for XIST generated by lncHUB2. For each gene set library, biological terms are ranked by the right-tailed p-value computed for the mean PCC between XIST and each gene set. luncHUB2 displays the top 15 terms from each gene set library. Black asterisks represent functions that are associated with XIST through a literature search.

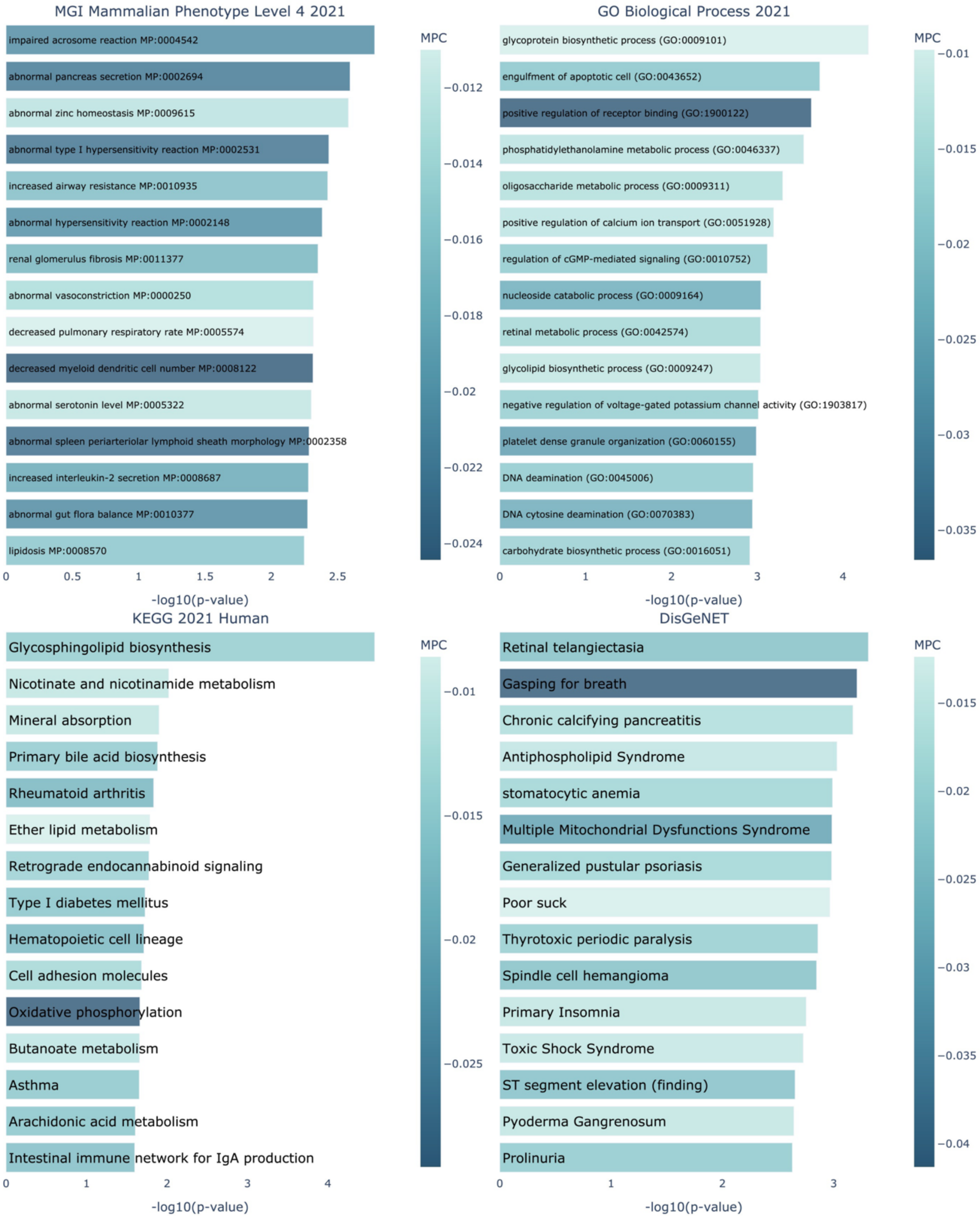

Figure S12. Predicted negatively correlated biological functions for XIST generated by lncHUB2. For each gene set library, biological terms are ranked by the left-tailed p-value for the mean PCC between XIST and each gene set. luncHUB2 displays the top 15 terms from each gene set library. These terms are predicted to be associated with XIST. Black asterisks represent functions that are associated with XIST through a literature search.

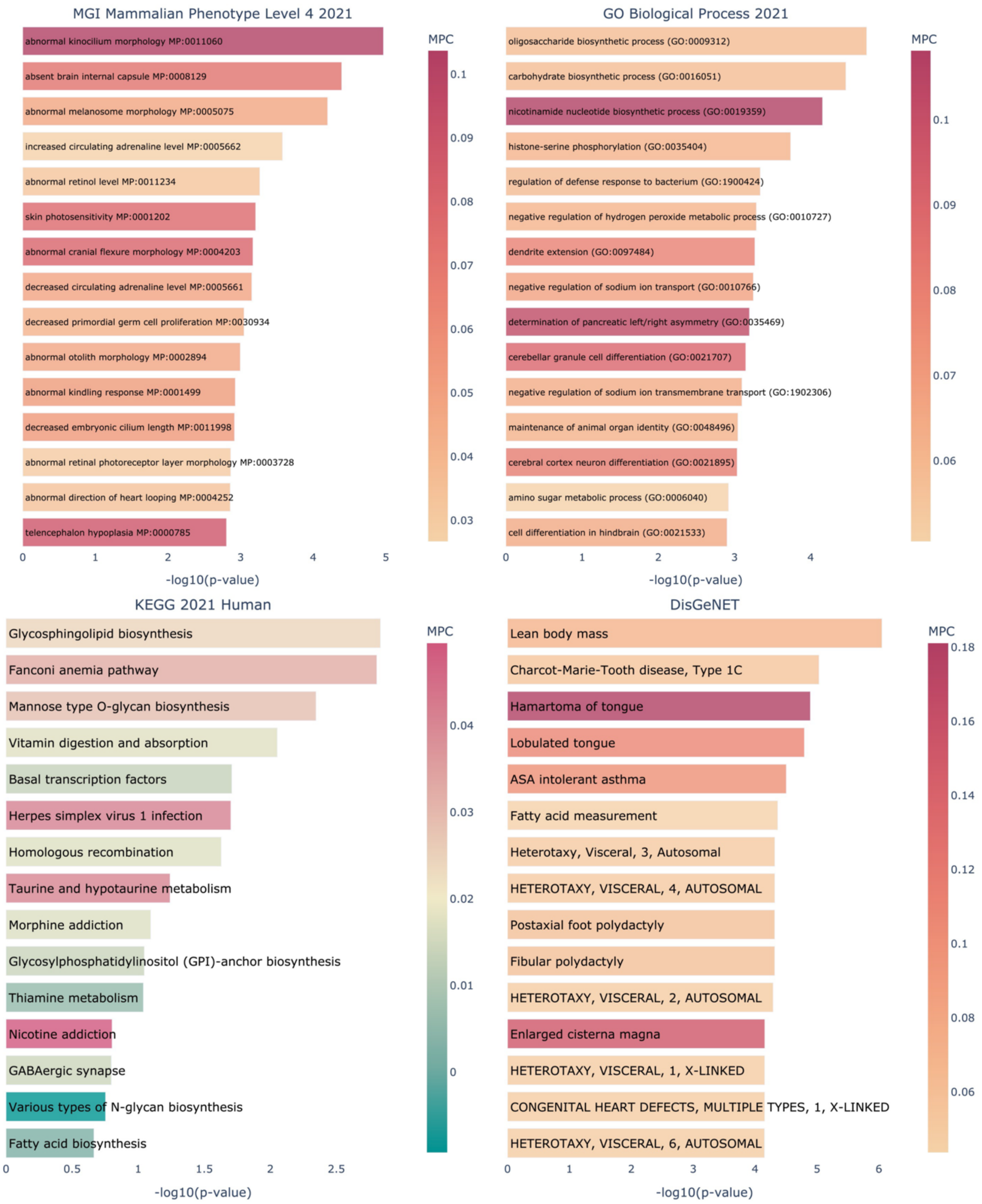

Figure S13. Predicted positively correlated biological functions for SAMMSON generated by lncHUB2. For each gene set library, biological terms are ranked by the right-tailed p-value computed for the mean PCC between SAMMSON and each gene set. lncHUB2 displays the top 15 terms from each gene set library. Black asterisks represent functions that are associated with SAMMSON through a literature search.

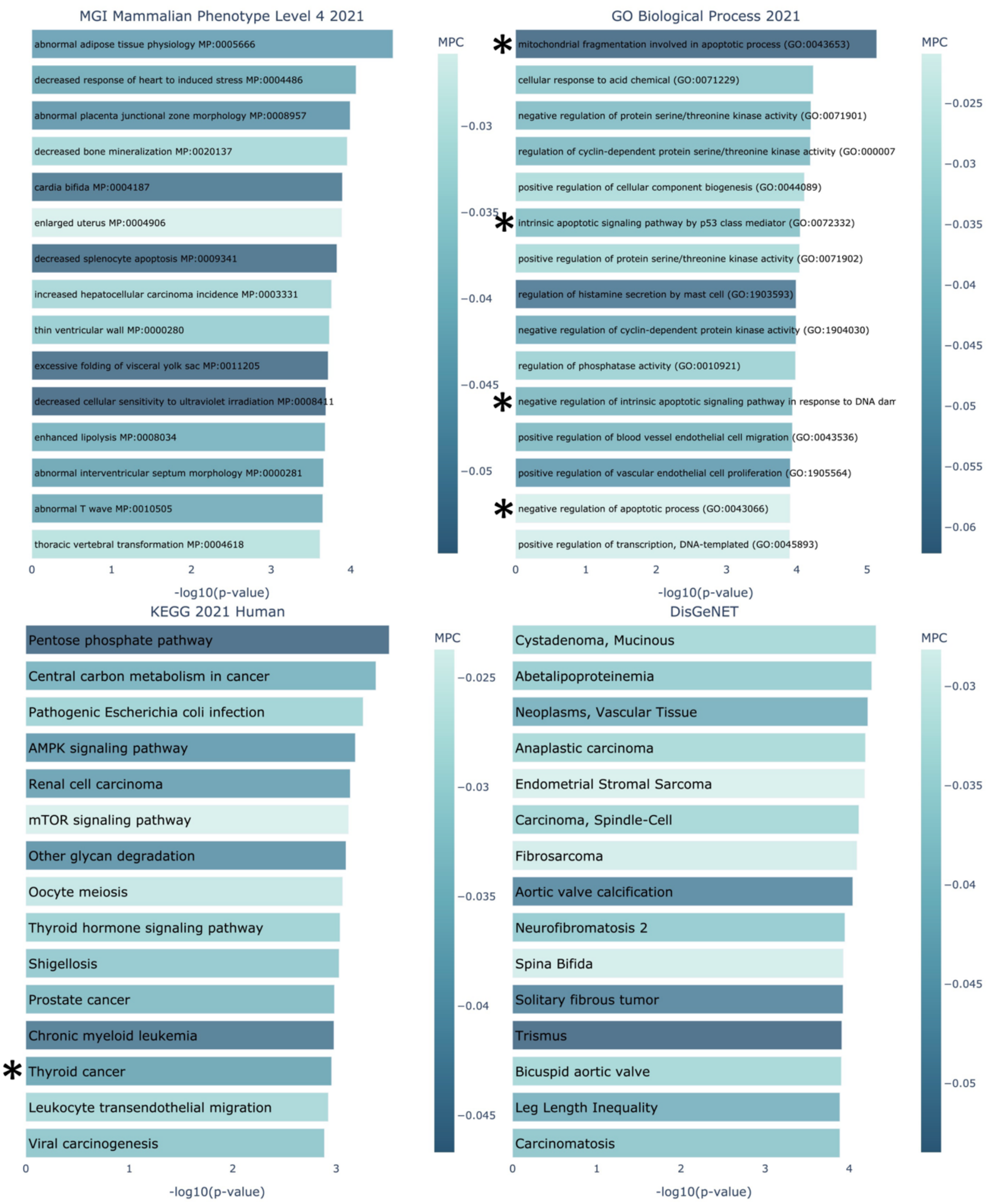

Figure S14. Predicted negatively correlated biological functions for SAMMSON generated by lncHUB2. For each gene set library, biological terms are ranked by the left-tailed p-value for the mean PCC between SAMMSON and each gene set. lncHUB2 displays the top 15 terms from each gene set library. Black asterisks represent functions that are associated with SAMMSON through a literature search.

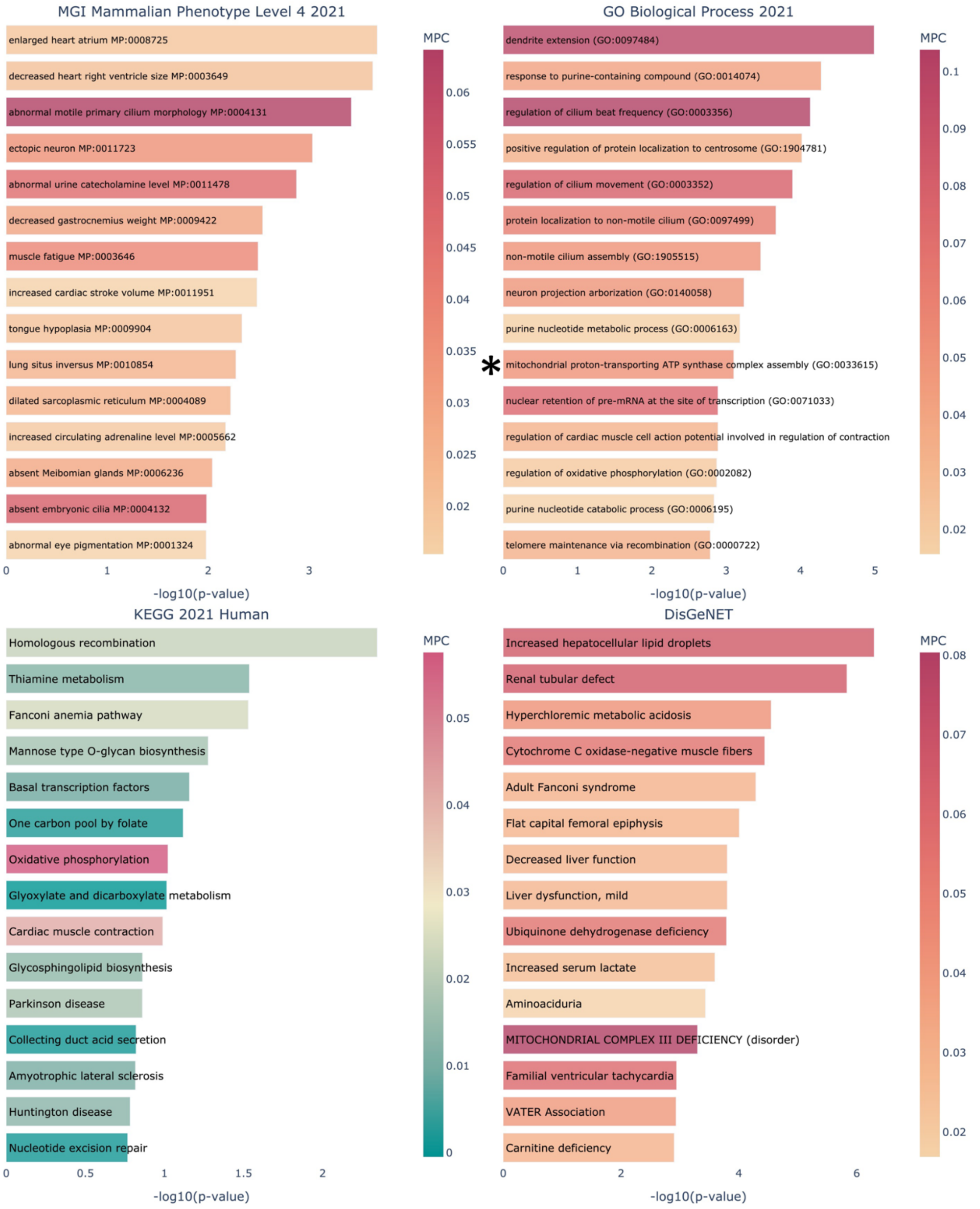

Figure S15. Predicted positively correlated biological functions for USP2-AS1 (glycoLINC) generated by lncHUB2. For each gene set library, biological terms are ranked by the right-tailed p-value computed for the mean PCC between USP2-AS1 and each gene set. luncHUB2 displays the top 15 terms from each gene set library. Black asterisks represent functions that are associated with USP2-AS1 through a literature search.

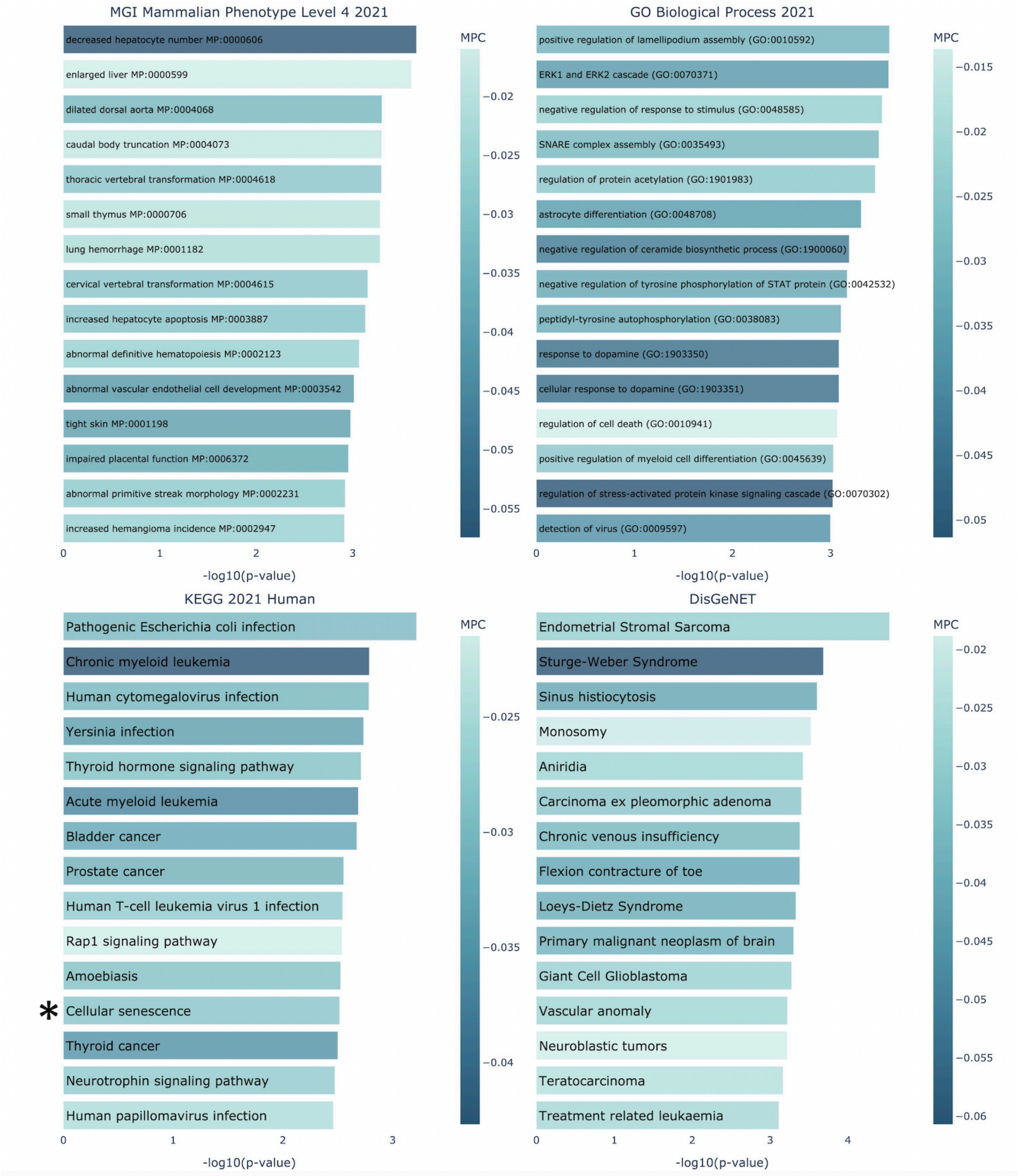

Figure S16. Predicted negatively correlated biological functions for USP2-AS1 (glycoLINC) generated by lncHUB2. For each gene set library, biological terms are ranked by the left-tailed p-value for the mean PCC between MEG3 and each gene set. luncHUB2 displays the top 15 terms from each gene set library. Black asterisks represent functions that are associated with USP2-AS1 through a literature search.
